# A comprehensive study of skin lesion segmentation and classification using deep learning techniques

**DOI:** 10.3389/frai.2026.1907266

**Published:** 2026-10-23

**Authors:** E. Babu, S. Murali

**Affiliations:** School of Computer Science and Engineering (SCOPE), Vellore Institute of Technology, Vellore, TN, India

**Keywords:** classification, convolutional neural network, deep learning, explainable artificial intelligence, segmentation, skin lesion analysis, U-net, vision transformer

## Abstract

Deep learning has become an important computational approach in dermatology because it enables automated segmentation and classification of skin lesions. Manual dermoscopic analysis is time-consuming and depends on specialist expertise, with potential inter-observer variability. There is a need to develop reliable computer-aided diagnostic tools. This structured narrative review covers journal papers published mainly from 2018 to 2026 on skin lesion analysis with focus on image preprocessing, benchmark datasets, conventional machine learning approaches, convolutional neural networks, U-Net architectures, transfer learning approaches, Vision Transformers, attention-based models, ensemble solutions, and hybrid segmentation–classification systems. Building on present reviews covering individual tasks as well as broader segmentation–classification pipelines, the present work provides a critical development-to-deployment analysis connecting dataset characteristics, preprocessing, segmentation, classification, evaluation, explainability, robustness, fairness, computational requirements, and clinical applicability. The paper describes widely used evaluation metrics and explains why results obtained across different datasets, partitions, and experimental setups should not be directly compared. Major open problems include class imbalance, data bias, domain shift, limited representation of diverse skin tones, inadequate external validation, computational requirements, and limited explainability of efficient models. Emerging trends such as self-supervised learning, Transformers, foundation models, explainable AI, federated learning, and multi-modal clinical data are reviewed as well. The review offers practical advice to researchers and clinicians on how to approach the preprocessing, segmentation, classification, and evaluation of skin lesions and highlights priorities in developing clinically reliable skin lesion analysis systems.

## 1 Introduction

The early diagnosis of skin cancer is associated with more favorable treatment options and increased survival rate. Nevertheless, dermoscopic analysis takes a long time to perform, demands great expertise and knowledge on behalf of a clinician and may involve inter-observer bias (Khattar and Kaur, 2022; Kassem et al., 2021). To overcome these disadvantages, computer-aided diagnosis systems were introduced and designed to automatically process the image and classify the skin lesions by means of artificial intelligence, deep learning, in particular (Cui et al., 2023). Convolutional neural networks gained popularity due to their ability to identify discriminating features in dermoscopic images. The neural network architectures such as U-Net, ResNet, DenseNet, EfficientNet, and their encoder-decoder analogs proved to be effective (Shetty et al., 2022; Al-Masni et al., 2020; Hasan et al., 2021).

Skin lesions, however, vary greatly in terms of size, shape, color, texture, pigmentation, and borders. Hair, rulers, bubbles, shadows, lighting conditions, and other such acquisition-based artifacts make automated analysis even less reliable. Recent research, therefore, focuses on improving diagnostic accuracy and generalization through various methods including preprocessing, augmentation, attention models, transfer learning, ensemble learning, post-processing, and segmentation–classification systems (Yang et al., 2021; Khattar and Kaur, 2022). Figure 1 summarizes the general workflow of automated skin lesion analysis from dataset acquisition through preprocessing, lesion segmentation, feature extraction, and diagnostic classification.

**Figure 1:**
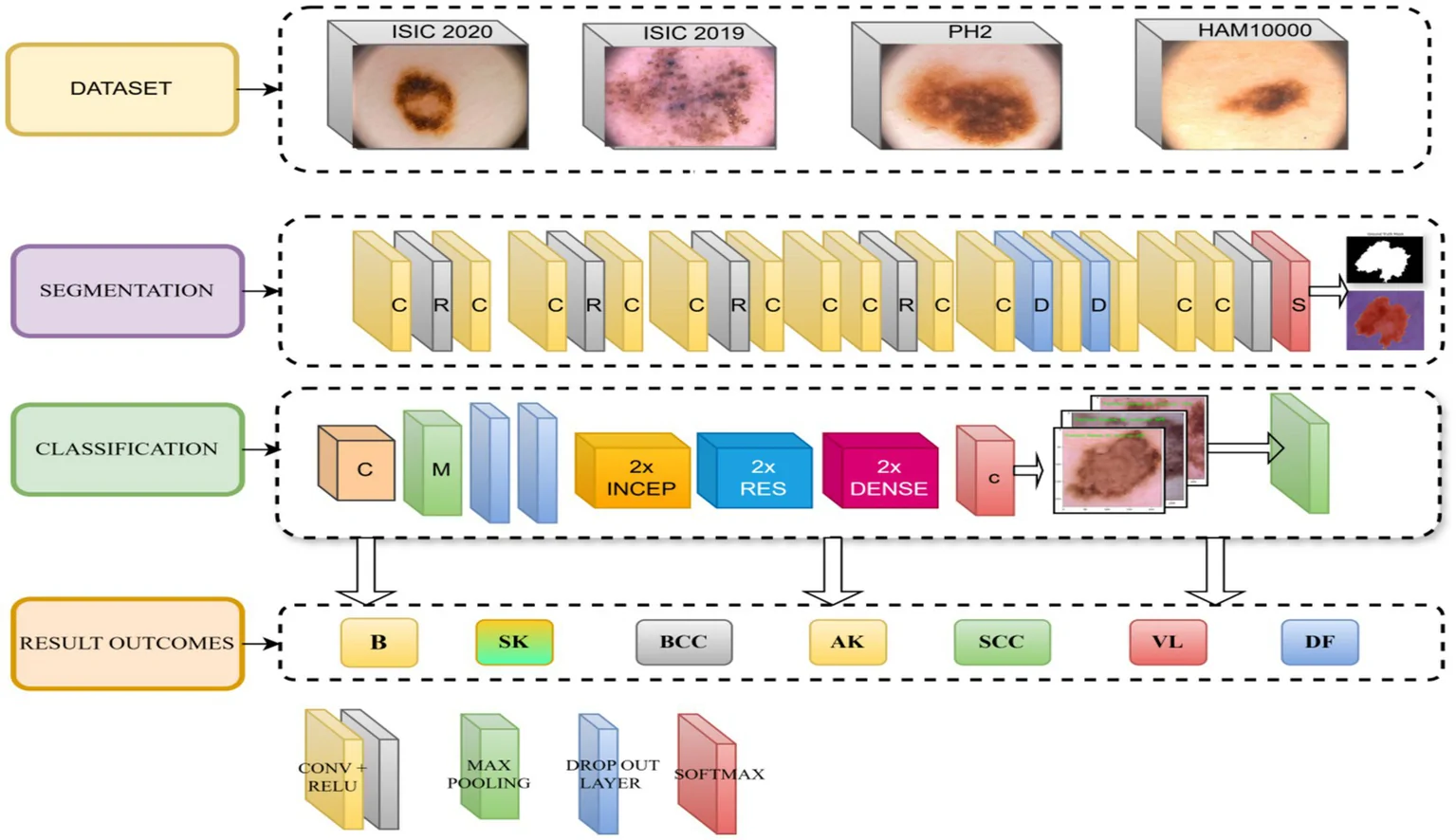
General workflow of an automated skin lesion analysis system, including dataset acquisition, preprocessing, lesion segmentation, feature extraction, diagnostic classification, and generation of predicted lesion categories.

The availability of large annotated databases has played a huge role in progress in automation of skin image analysis. The archive of International Skin Imaging Collaboration comprises dermoscopic images gathered from multiple data sources and has enabled a number of segmentation and classification competitions. The HAM10000 database consists of 10,015 multi-source dermoscopic images that correspond to seven types of pigmented skin lesions and is utilized for testing classification systems (Tschandl et al., 2018). There are other databases that include PH^2^ and Derm7pt, where there are various differences in acquisition parameters, diagnostic categories, clinical metadata, and lesion labels.

However, a number of issues exist, which need to be addressed. The variability of the lesions’ appearance complicates segmentation and classification. Artifacts and acquisition differences can make a model learn irrelevant properties of the images. Class imbalance can cause a classifier to give priority to benign lesions, which occur more frequently than others. Lack of generalization to external datasets, underrepresentation of darker skin tones, inaccurate estimates of uncertainties, and inability to interpret deep learning predictions pose serious challenges (Nguyen et al., 2022; Singh et al., 2022; Khattar and Kaur, 2022).

The current paper offers an extensive and critical overview of deep learning methods applied to skin lesion segmentation and classification tasks. The basic theoretical aspects, datasets, pre-processing and augmentation techniques, machine learning techniques, CNNs, U-Nets, transfer learning, vision transformers, ensembles of methods, self-supervised learning, multimodal learning, foundation models, explainable artificial intelligence, and evaluation criteria are examined in this paper. In addition, the drawbacks of the existing approaches and research directions are discussed in this review.

### 1.1 Scope and unique aspects of the current review

The current paper is developed as a structured review of literature on automatic skin lesion segmentation and classification. The scope of the paper includes dermoscopic and clinically relevant skin lesions, publicly available datasets, image pre-processing, data augmentation, machine learning models, CNNs, U-Nets, transfer learning, attention mechanisms, vision transformers, ensembles of models, self-supervised learning, multimodal learning, explainable artificial intelligence, and integrated segmentation–classification approaches.

Traditional image processing and machine learning techniques are also included in the discussion to provide context on the history of the field and to make comparisons with current deep learning methodologies. More attention is paid to techniques that assist in skin lesion localization, diagnosis classification, robustness, interpretability, computational efficiency, or clinical translation. Other criteria considered include sensitivity, specificity, precision, F1-score, balanced accuracy, Area Under the Receiver Operating Characteristic Curve, Dice coefficient, Intersection over Union, boundary accuracy, calibration, computational efficiency, external generalization, and clinical applicability.

Other review papers have already offered important information concerning skin lesions classification, segmentation, or deep learning in general (Mohd Stofa et al., 2021; Kassem et al., 2021; Khattar and Kaur, 2022). Nevertheless, earlier reviews have often emphasized particular analytical tasks, architecture families, benchmark datasets, or specific methodological themes, although more recent reviews have increasingly addressed broader segmentation–classification pipelines.

First, this literature review examines the impact of preprocessing on feature representation, the influence of segmentation on classification, and the relations between architecture, dataset size, class imbalance, computational resources, explainability, and clinical constraints. Recent developments on data augmentation, segmentation using transformer networks, self-supervised learning, foundation models for medicine, multimodal approaches, and explainable skin lesion classification have been reviewed in this paper (Garcea et al., 2023; Chen et al., 2024; Ma et al., 2024; Wang Y. et al., 2023; Wang H. et al., 2025; Ruga et al., 2026).

Recent literature reviews have been becoming more and more exhaustive in terms of the coverage of AI-driven skin lesion analysis. In particular, Hasan et al. (2023) have reviewed 594 studies on skin lesion segmentation and classification, examining the literature in terms of datasets, preprocessing, augmentation, model configurations, training methods, and evaluation metrics. It is important that this paper is an extensive overview of computer-assisted methods of skin lesion analysis, nevertheless, the reviewed literature represents the papers published by 2022.

Further literature reviews have become increasingly extensive due to taking into account new developments in deep learning and computer vision. Ray et al. (2024) have reviewed 107 publications concerning skin cancer classification, analyzing conventional machine learning, CNNs, transfer learning, attention mechanisms, ensembles, generative models, Vision Transformers, classification guided by segmentation, hybrid approaches, and multimodal systems. Zareen et al. (2025) have reviewed the pipeline of computer-assisted diagnosis of skin lesions from data preparation and lesion segmentation to feature extraction, feature selection, and final classification.

Also, reviews focusing on segmentation have become much more elaborated. Sharma et al. (2025) carried out a systematic review of skin lesion segmentation algorithms published during the period from 2018 to 2025 with regard to the use of data sets, data pre-processing and augmentation, architecture, loss function, hyper-parameters, and evaluation metrics. Such works provide important methodology summaries, yet, their main focus is on segmentation itself, not the relations between lesion localization, classification, validation, and clinical implementation.

Further literature of 2026 shows that the reviewing sphere keeps developing actively, Saleh et al. (2026) provided a systematic review on deep learning methods in skin cancer segmentation and classification, having selected 77 experimental works out of 540 and showing the predominance of CNNs for classification and U-Net-based models for segmentation. Bala et al. (2026) also performed a systematic review of image-processing approaches in skin lesion detection, including segmentation and classification techniques and open-source data sets. Such recent works prove the fast development of the sphere and show that segmentation and classification are becoming an increasingly important part of computer-assisted diagnostics.

Concurrently, recent research has also drawn attention to constraints beyond performance in architecture. For instance, Tjiu and Lu (2025) conducted a systematic study on the issues of equity and generalizability of artificial intelligence technology in skin lesion diagnosis using clinical images, dermoscopic images, and smartphone images. The study focused on evaluating the diagnostic performance using various imaging modes and contexts, emphasizing the need to focus on external validity and equitable performance. Such considerations are critical since performance based on benchmark datasets commonly used may not reflect performance based on different conditions of acquisition, different populations, skin pigmentation, different devices, and clinical context. Table 1 compares recent reviews according to review type, literature time span, number of publications or studies reported, principal topic coverage, and the objective positioning of the present review.

**Table 1:** Comparison of recent review studies and objective positioning of the present review.

Review	Year	Review type	Literature time span	Number reported/reviewed	Main topic coverage	Objective positioning
Hasan et al.	2023	Comprehensive survey/review	2011–2022	594 publications	Skin lesion segmentation and classification; datasets, preprocessing, augmentation, architectures, training, and evaluation	Broad coverage of segmentation and classification; the present review additionally examines validation, robustness, fairness, explainability, external validation, computational feasibility, and clinical translation
Ray et al.	2024	Survey	Previous 18 years	107 research papers	Skin cancer classification; machine learning, CNNs, transfer learning, attention, ensembles, GANs, Vision Transformers, segmentation-guided classification, hybrid and multimodal methods	Primarily classification-focused; the present review considers segmentation and classification together with validation, robustness, explainability, and clinical translation
Zareen et al.	2025	Review article	Previous 6 years	Not specified as a formal inclusion count	Computer-aided diagnosis; data preparation, lesion segmentation, feature extraction, feature selection, and classification	Broad diagnostic-pipeline coverage; the present review additionally considers evidence quality, robustness, fairness, explainability, external validation, and deployment
Sharma et al.	2025	Systematic review	2018–2025	Not specified	Skin lesion segmentation; datasets, preprocessing, augmentation, model configurations, losses, hyperparameters, and evaluation metrics	Detailed segmentation-focused coverage; the present review connects segmentation with classification, validation, robustness, and clinical applicability
Tjiu and Lu	2025	Systematic review and meta-analysis	Inception–31 October 2025	18 studies	Equity and generalizability of AI-based skin-lesion diagnosis using clinical, dermoscopic, and smartphone images; diagnostic accuracy and fairness	Focuses specifically on equity, generalizability, and external validity; the present review integrates these concerns with methodological choices across the broader analysis pipeline
Saleh et al.	2026	Systematic review	2014–2024	77 experimental studies from 540 records	Deep learning for skin cancer segmentation and classification; CNN-based classification, U-Net-based segmentation, hybrid and ensemble approaches, and risk of bias	Provides recent systematic evidence on segmentation and classification; the present review additionally emphasizes methodological dependencies, validation, computational feasibility, explainability, robustness, fairness, and clinical translation
Bala et al.	2026	Systematic review	2012–2025	63 records finally selected from 157 manually retrieved papers	Skin lesion detection; image preprocessing, segmentation, feature extraction/selection, classification, hybrid approaches, and public datasets	Provides recent systematic coverage of skin lesion detection; the present review extends the analysis toward development-to-deployment trade-offs, external validation, robustness, fairness, explainability, and clinical translation
Present Study	2026	Structured narrative review	Primarily 2018–2026	No fixed systematic-review count	Datasets, preprocessing, segmentation, classification, evaluation, explainability, robustness, fairness, external validation, self-supervised learning, multimodal learning, foundation models, and clinical translation	Provides a critical development-to-deployment synthesis linking methodological choices with evidence quality, generalizability, computational feasibility, interpretability, fairness, and clinical applicability

Recent reviews have progressively expanded the coverage of skin lesion analysis, but they differ substantially in review type, literature time span, methodological emphasis, and topic coverage. Hasan et al. (2023) provided broad coverage of skin lesion segmentation and classification, while Ray et al. (2024) focused primarily on skin cancer classification. Zareen et al. (2025) examined the broader computer-aided diagnostic pipeline, whereas Sharma et al. (2025) focused specifically on segmentation. Tjiu and Lu (2025) emphasized equity and generalizability, particularly external validity across imaging modalities and populations. More recent systematic reviews by Saleh et al. (2026) and Bala et al. (2026) further expanded the evidence base for segmentation, classification, and detection. The present review differs in its structured narrative approach and development-to-deployment perspective, linking datasets, preprocessing, segmentation, classification, validation, explainability, robustness, fairness, computational requirements, and clinical translation rather than focusing on a single task or methodological family.

This perspective helps identify where reported improvements are supported by stronger evidence and where apparent gains may depend on dataset characteristics, validation protocols, or increased computational complexity.

### 1.2 Literature search and selection criteria

Recent primary studies were also considered to capture emerging directions such as multimodal fusion of dermoscopic images and clinical metadata, lightweight Vision Transformer architectures for smartphone-based diagnosis, YOLO-based lesion detection, and attention-based Transformer classification (Wang S. et al., 2025). A structured literature search was performed via Scopus, Web of Science, PubMed, IEEE Xplore, ScienceDirect, SpringerLink, and Google Scholar using individual and combinations of the following keywords: “skin lesion,” “skin cancer,” “dermoscopy,” “deep learning,” “machine learning,” “segmentation,” “classification,” “CNN,” “U-Net,” “transfer learning,” “Vision Transformer,” “explainable artificial intelligence,” “self-supervised learning,” “foundation model,” and “computer-aided diagnosis.” The final literature search was conducted on 21 September 2026.

The literature review mainly focused on journal articles published from 2018 to 2026. Early works were retained if needed to provide background for the emergence of data preparation, preprocessing, segmentation, classification, and evaluation approaches. Current journal articles were retained to incorporate the progress in augmentation, Transformer-based segmentation, self-supervised learning, multimodal classification, medical foundation models, and explainable artificial intelligence.

Current augmentation research included medical image augmentation research, mixing-based augmentation, interpretability-aware augmentation, and diffusion-based synthetic image generation (Garcea et al., 2023; Lewy and Mańdziuk, 2023; Singh et al., 2021; Liu et al., 2025; Alimisis et al., 2025). Current Transformer-based segmentation research included TransUNet and current architecture designs specifically for skin lesions with modeling global context, statistical texture, and structural information (Chen et al., 2024; Xin et al., 2024; Xu et al., 2024; Wang Z. et al., 2025).

Representatives of medical foundation models and self-supervised approaches included research on MedSAM, self-supervised knowledge distillation, and multimodal skin lesion learning (Ma et al., 2024; Wang Y. et al., 2023; Wang H. et al., 2025). Explainability of models was studied using recent skin lesion frameworks with the use of methods like Grad-CAM and SHAP (Ruga et al., 2026; Fiaz et al., 2025).

The inclusion criteria for studies were the following:

1. The article contained the proposal, assessment, or comparison of a technique for skin lesion segmentation/classification.
2. The work analyzed preprocessing, augmentation, feature extraction, class imbalance, transfer learning, explainability, computational deployment, or clinical translation, as applied to the problem of skin lesions.
3. Methods, dataset, model architecture, training, evaluation measures, or comparative performance results were described sufficiently.
4. There were issues regarding generalization, robustness, fairness, clinical interpretability, and deployment of the technique discussed.
5. The publication was an article, and the used data was published and could be included in a review article.

Studies with duplicate data, papers outside the scope of skin lesion imaging, those without adequate methodological contribution, conference papers, preprint papers, and studies that only addressed other applications such as anatomy and agriculture imaging were eliminated from the primary body of evidence. General medical imaging journal articles were included in the study only if they contributed important methodological knowledge related to augmentation, Transformer segmentation, foundation models, self-supervised learning, or explainability in regard to skin lesion analysis.

Studies identified through the database searches were screened in two stages. First, the titles and abstracts of the identified publications were screened to exclude studies that were clearly outside the scope of the review. Second, the full texts of potentially relevant publications were assessed against the predefined inclusion and exclusion criteria. Reference lists of relevant review and primary studies were also examined to identify additional studies directly relevant to the objectives of the review.

For the comparative tables, representative studies were selected based on their direct relevance to the objectives of the review, methodological diversity, use of established or clearly described datasets, sufficient reporting of methods and evaluation, and their ability to illustrate important methodological contributions, limitations, practical trade-offs, or emerging research directions. Recent studies representing emerging developments, including Transformer-based methods, self-supervised learning, multimodal learning, foundation models, and explainable artificial intelligence, were also considered.

Studies were not selected solely because they reported the highest accuracy, Dice, IoU, or AUC, because performance values obtained using different datasets, data partitions, class distributions, preprocessing procedures, and evaluation protocols are not directly comparable. Accordingly, Tables 1–5 present representative examples of the literature rather than exhaustive listings or performance rankings.

**Table 2:** Representative CNN-based, hybrid, self-supervised and Transformer-based approaches for skin lesion analysis and their methodological trade-offs.

Study	Dataset and task	Architecture / Approach	Key reported result	Critical interpretation
Shetty et al. (2022)	HAM10000, seven-class lesion classification	Customized CNN compared with conventional machine-learning classifiers, using data augmentation and k-fold cross-validation	CNN achieved 95.18% accuracy.	Demonstrates the effectiveness of a compact CNN for multiclass classification. However, the evaluation used a selected and augmented dataset configuration, and external validation was not reported.
Al-Masni et al. (2020)	ISIC-2016, ISIC-2017 and ISIC-2018, lesion segmentation followed by classification	Full-resolution CNN segmentation followed by Inception-v3, ResNet-50, Inception-ResNet-v2 and DenseNet-201	ResNet-50 achieved 89.28% weighted accuracy on ISIC-2018, segmentation also improved classification performance on ISIC-2016.	Demonstrates the potential benefit of segmentation-guided classification by reducing background interference. However, segmentation errors can propagate to the classification stage.
Hasan et al. (2021)	ISIC-2016 and ISIC-2017, concurrent lesion detection and recognition	Dermo-DOCTOR with dual encoders, channel-wise feature fusion, lesion-mask decoder and classification outputs	Mean IoU of 85.0 and 80.0% and recognition AUC of 0.98 and 0.91 on ISIC-2016 and ISIC-2017, respectively.	Demonstrates the benefit of joint detection and recognition. However, the dual-encoder and feature-fusion design increases architectural complexity and requires careful cross-dataset evaluation.
Kassem et al. (2020)	ISIC-2019, eight-class lesion classification	Modified GoogleNet with transfer learning and class balancing	94.92% accuracy, 79.8% sensitivity, 97.0% specificity and 80.36% precision.	Shows the importance of addressing class imbalance. High overall accuracy should be interpreted together with class-sensitive measures, majority-class reduction may also discard useful training information.
Khan et al. (2021)	ISBI-2016, ISBI-2017, ISIC-2018 and PH2 for segmentation, HAM10000 for seven-class classification	Deep Saliency Segmentation using a custom 10-layer CNN, ResNet101/DenseNet201 feature extraction, improved moth flame optimization (IMFO), multiset maximum correlation analysis (MMCA), and KELM classification	Segmentation accuracies of 95.38%, 95.79%, 92.69 and 98.70% on ISBI-2016, ISBI-2017, ISIC-2018 and PH2, respectively, 90.67% classification accuracy on HAM10000.	Integrates lesion segmentation, deep feature extraction, feature selection, feature fusion and classification, but the multiple processing stages increase computational complexity and reduce end-to-end simplicity.
Wang H. et al. (2023)	HAM10000, seven-class lesion classification, additionally evaluated on a clinical skin-lesion dataset	Double-branch network (DBN) with feature extraction and fusion branches	On HAM10000, DBN achieved 91.58% accuracy, 90.89% balanced accuracy, 85.03% F1-score and 0.985 AUC.	Demonstrates the value of multi-branch feature fusion. However, the authors reported weaker recognition of minority categories such as melanoma and SCC, illustrating the continuing effect of class imbalance.
Wang Y. et al. (2023)	ISIC-2019, eight-class skin-disease classification	SSD-KD, combining self-supervised relational learning and knowledge distillation, MobileNetV2 student network	The distilled MobileNetV2 achieved up to 85% accuracy while substantially reducing model size and computational requirements.	Represents a shift toward lightweight and self-supervised learning. Knowledge distillation improves computational efficiency, but compression must be evaluated carefully for sensitivity to minority and malignant categories.
Xu et al. (2024)	ISIC-2018 and two additional public skin-lesion datasets, lesion segmentation	SkinFormer, a Transformer architecture incorporating statistical texture representation through kurtosis-guided statistical counting and texture-fusion/enhancement modules	Achieved 93.2% Dice on ISIC-2018 and reported superior performance against compared state-of-the-art methods across three public datasets.	Demonstrates the move from purely convolutional feature extraction toward Transformer-based global context and statistical texture modeling. The additional architectural components may increase computational and implementation requirements.

**Table 3:** Representative preprocessing and augmentation studies and their methodological trade-offs.

Study	Main preprocessing or augmentation focus	Application	Main contribution	Important limitation or consideration
Garcia-Arroyo and Garcia-Zapirain (2019)	Histogram thresholding and fuzzy pixel classification	Skin lesion segmentation	Demonstrated the continued usefulness of color and intensity information for lesion-region separation	Performance may be sensitive to illumination, contrast, and threshold-related assumptions
Nida et al. (2019)	Region-based CNN localization combined with Fuzzy C-Means clustering	Melanoma lesion detection and segmentation	Combined learned region localization with clustering-based boundary delineation	Clustering may remain sensitive to initialization, color variation, and artifacts
Mahbod et al. (2019)	Multi-scale image representation and fusion of fine-tuned deep features	Skin lesion classification	Demonstrated that complementary representations extracted at different scales can improve diagnostic feature learning	Multi-scale processing increases computational cost and may require careful normalization
Ashraf et al. (2022)	Training and test-time augmentation with Conditional Random Field refinement	Melanoma segmentation	Illustrated how augmentation and post-processing can improve segmentation robustness and boundary consistency	Test-time augmentation and CRF refinement increase inference complexity
Bansal and Sridhar (2024)	GAN-based image inpainting	Hair and artifact correction in skin lesion images	Investigated generative reconstruction of occluded lesion regions	Generated content may introduce artificial texture and requires clinical validation
Garcea et al. (2023)	Conventional and generative augmentation methods	Medical image analysis	Provided a systematic synthesis of augmentation strategies, benefits, and evaluation issues	Augmentation effectiveness depends on task, dataset, and clinical plausibility
Lewy and Mańdziuk (2023)	MixUp, CutMix, and related mixing strategies	Image classification and representation learning	Compared mixing-based augmentation methods and their regularization characteristics	Mixed images may not correspond to realistic clinical samples
Singh et al. (2021)	Augmentation within few-shot meta-learning	Limited-data medical image classification	Demonstrated the role of augmentation in improving learning from small medical datasets	Performance may depend strongly on task similarity and episode construction
Liu et al. (2025)	Relationship between augmentation and model explainability	Medical image classification	Examined how augmentation can change both prediction and explanation behavior	Better predictive performance does not necessarily imply more stable explanations
Alimisis et al. (2025)	Diffusion-based image augmentation	Synthetic-image generation	Reviewed diffusion models, evaluation criteria, and future augmentation directions	Computational demand, memorization, clinical validity, and bias require careful assessment

**Table 4:** Representative skin lesion segmentation studies and their principal methodological trade-offs.

Study	Dataset and validation strategy	Segmentation approach	Main contribution	Principal consideration
Al-Masni et al. (2018)	ISBI 2017 + PH2; cross-dataset testing	Deep full-resolution convolutional network	Preserved spatial resolution for pixel-level lesion segmentation	Deep models remain dependent on annotation quality and dataset diversity
Garcia-Arroyo and Garcia-Zapirain (2019)	ISBI 2016/2017; challenge test sets	Histogram thresholding and fuzzy pixel classification	Demonstrated a transparent conventional segmentation strategy	Sensitive to intensity, color, and illumination variation
Nida et al. (2019)	Melanoma dermoscopic images	Deep region-based CNN with Fuzzy C-Means	Combined learned lesion localization with clustering-based delineation	Clustering depends on initialization and lesion–skin distribution
Hasan et al. (2020)	ISIC-2017 + PH2; cross-dataset testing	DSNet CNN segmentation architecture	Developed an automatic network for dermoscopic lesion segmentation	External generalization requires evaluation across datasets
Xie et al. (2020)	Dermoscopic lesion images	High-resolution CNN	Maintained high-resolution representations for detailed segmentation	High-resolution processing may increase computation and memory
Liu et al. (2021)	Skin lesion datasets	Deep segmentation with an auxiliary task	Used additional supervision to improve lesion representation	Benefit depends on task compatibility and loss weighting
Ashraf et al. (2022)	ISIC-2016/2017; combined and individual test sets	Deep segmentation, test-time augmentation, and CRF refinement	Improved robustness and mask consistency through combined refinement	Test-time augmentation and CRF increase inference complexity
Alahmadi (2022)	Dermoscopic lesion datasets	Multi-scale Attention U-Net	Combined attention and multi-scale representation for boundary localization	Additional modules require ablation and computational analysis
Wang H. et al. (2023)	Skin disease segmentation	Transformer–MLP hybrid encoder	Combined local and global feature representations	Hybrid complexity may affect reproducibility and deployment
Li and Zhang (2024)	HAM10000 and additional dermoscopic data	Weakly supervised SG-MIAN	Reduced dependence on pixel-level masks using image-level supervision	Weak supervision may produce less precise boundaries than full supervision
Chen et al. (2024)	Medical image segmentation datasets	TransUNet	Combined Transformer global context with U-Net localization	Requires greater computation and commonly benefits from Pre-training
Xin et al. (2024)	ISIC-2016/2017/2018	Transformer-guided self-adaptive multi-scale network	Modeled lesions of varying size using multi-scale and cross-attention features	Complexity and external robustness require careful evaluation
Xu et al. (2024)	multiple public datasets	SkinFormer	Integrated statistical texture and structural Transformer representations	Domain-specific design should be tested across devices and populations
Ma et al. (2024)	medical datasets including dermoscopy; internal/external validation	MedSAM	Adapted promptable foundation-model segmentation to medical images	Independent external dermoscopic validation remains necessary for deployment on datasets distinct from those used during foundation-model development
Wang Z. et al. (2025)	validation details not specified	Superpixel Embedded Transformer	Used structure-aware superpixel tokens for lesion segmentation	Computational requirements and cross-dataset generalization require assessment

**Table 5:** Representative skin lesion classification studies and their principal trade-offs.

Existing study	Dataset and validation strategy	Classification approach	Main contribution	Principal consideration
Shetty et al. (2022)	HAM10000 dermoscopic images; 10-fold cross-validation	Machine learning and CNN classification	Compared conventional classifiers and learned CNN representations	Internal accuracy should be supplemented by class-wise and external evaluation
Kassem et al. (2020)	ISIC-2019; 80% training, 10% validation, and 10% testing	Deep CNN and transfer learning for eight lesion classes	Demonstrated multiclass transfer-learning classification	Class imbalance and dataset-specific performance require careful interpretation
Mahbod et al. (2019)	ISIC-2016 and ISIC-2017 training/validation data; 600-image ISIC-2017 challenge test set	Fusion of fine-tuned deep features	Combined complementary pre-trained representations	Feature fusion increases computational and storage requirements
Mahbod et al. (2020)	ISIC-2018 classification challenge test set; held-out test-set evaluation	Multi-scale and multi-network ensemble	Used scale and network diversity to improve classification	Ensemble complexity may restrict real-time deployment
Nguyen et al. (2022)	HAM10000; 90% training, 10% validation, plus a separate 857-image test set	CNN with soft attention	Addressed lesion focus under class imbalance	Attention should be validated for stability and clinical relevance
Cirrincione et al. (2023)	ISIC-2017; 2,000 training images, 150 validation images, and 600 test images; held-out test-set evaluation	Vision Transformer (ViT)-based classification	Applied a Transformer-based architecture to skin lesion classification and evaluated different ViT configurations for melanoma detection	Transformer models require adequate data, computational resources, and independent external validation to assess generalization beyond the ISIC-2017 benchmark
Qian et al. (2022)	HAM10000; validation/split details not specified in the reported study	CNN with multi-scale attention and class-specific loss weighting	Combined attention with imbalance-aware optimization	Performance depends on loss-weight selection and dataset composition
Popescu et al. (2022)	HAM10000; 70% training and 30% validation on the development portion, with a separate testing portion	Multiple-network ensemble	Used collective predictions from several neural networks	Higher inference cost and reduced interpretability
Alam et al. (2022)	HAM10000; internal validation and test-set evaluation	S2C-DeLeNet integrated segmentation–classification model	Transferred information between segmentation and classification	Segmentation errors may influence diagnostic classification
Wang H. et al. (2023)	CSLI and PAD-UFES-20; 8:1:1 train/validation/test split	Double-branch classification network	Combined complementary feature branches	Multi-branch systems require component-wise ablation
Wang Y. et al. (2023)	ISIC-2019; evaluation/split details not specified in the reported study	Self-supervised diverse knowledge distillation	Developed a lightweight student classifier using teacher knowledge	Compression must preserve malignant-class sensitivity
Wang H. et al. (2025)	Derm7pt; 413 training, 203 validation, and 395 testing studies	Self-supervised multimodal classification	Learned complementary information from two image modalities	Missing modalities and unequal image quality remain challenges
Ruga et al. (2026)	HAM10000, ISIC, and MED-NODE; HAM10000 evaluated using a 70/15/15 train/validation/test split, with evaluation also performed across ISIC and MED-NODE	MultiExCam explainable ensemble framework	Combined deep, handcrafted, ensemble, Grad-CAM, and SHAP information	Multiple components increase computational and interpretive complexity
Fiaz et al. (2025)	HAM10000; 60% training, 20% validation, and 20% testing	Explainable hybrid segmentation and multiclass classification	Integrated lesion localization, classification, and Grad-CAM	External and prospective validation remain necessary

Because this is a structured narrative review rather than a prospectively registered systematic review, a PRISMA-style record count was not maintained during the original search. Exact numbers of records retrieved, screened, and finally included are therefore not available retrospectively without reconstructing the searches; estimated counts have not been introduced because they could be misleading. Numerical results from individual studies were therefore qualitatively synthesized rather than statistically pooled, and meta-analysis was not considered appropriate because of substantial differences among studies in dataset structure, lesion classes, image resolution, training/testing split, augmentation strategies, loss function, architecture configuration, and evaluation metrics.

### 1.3 Main contributions

The main contributions of the present review are as follows:

1. First, it provides a critical development-to-deployment analysis of datasets, preprocessing, segmentation, classification, evaluation, explainability, robustness, fairness, and clinical translation within skin lesion analysis.
2. Second, it compares classical image processing approaches, traditional machine-learning classifiers, CNNs, U-Nets, vision transformers, transfer-learning approaches, ensemble learning and hybrid methods.
3. Third, it clarifies why certain preprocessing and data augmentation methods need to be chosen depending on artifacts, properties of specific dataset, downstream task, and type of architecture.
4. Fourth, it considers recent advances in advanced data augmentation, Transformer-based segmentation, medical foundation models, self-supervised learning, multimodal information processing, and explainable artificial intelligence (Garcea et al., 2023; Chen et al., 2024; Ma et al., 2024; Wang Y. et al., 2023; Wang H. et al., 2025; Ruga et al., 2026).
5. Fifth, it assesses not only accuracy but also robustness, class imbalance, computational complexity, interpretability, calibration, external validation, and clinical translation of considered approaches.
6. Sixth, it identifies methodological and clinical challenges by examining the trade-offs among performance, robustness, generalizability, computational requirements, interpretability, fairness, and clinical applicability.

Across the reviewed literature, reported improvements are frequently associated with differences in dataset composition, preprocessing, augmentation, data partitioning, class distribution, architectural complexity, and evaluation protocols. Consequently, the present review interprets reported performance in relation to these methodological conditions rather than treating individual metrics as directly comparable evidence of superiority. This approach allows the subsequent sections to examine not only what methods achieve, but also under which experimental and deployment conditions their reported benefits may be reproducible or clinically meaningful.

## 2 Basic characteristics of skin lesions

The basic features of skin lesions are important for developing accurate skin lesion segmentation and classification systems. Skin lesions are very different concerning their morphology, pigmentation, texture, border structure, anatomical position, and clinical importance. All those differences may cause problems in the visual diagnosis, not only for professionals but for the automatic image analysis as well. In the process of skin lesion analysis, the lesion is detected, its visual properties are determined, and the lesion is classified into the corresponding category. Automatic systems usually analyze the asymmetry, border structure, coloration, diameter, texture, vascular structures, and other dermoscopic structures. Machine learning models try to find those features directly from dermoscopic and clinical images without using manually designed features (Shetty et al., 2022; Kassem et al., 2021).

### 2.1 Types of skin lesions

Skin lesions can be broadly categorized as benign, premalignant, or malignant. Common categories represented in dermoscopic datasets include melanoma, melanocytic nevus, basal cell carcinoma (BCC), squamous cell carcinoma (SCC), actinic keratosis (AK), benign keratosis (BKL), dermatofibroma (DF), and vascular lesions. Their visual similarity, heterogeneous pigmentation, irregular borders, and variable morphology make automated differentiation challenging (Tschandl et al., 2018; Kassem et al., 2021).

Melanoma is a malignant tumor originating from melanocytes and may exhibit asymmetry, irregular borders, multiple colors, atypical pigment networks, and heterogeneous internal structures. Melanocytic nevi are generally benign but atypical nevi may resemble melanoma. BCC is a non-melanoma skin cancer commonly occurring on sun-exposed skin and may show arborizing vessels, blue-gray structures, ulceration, or shiny white areas. SCC is a malignant keratinocytic tumor that may present with scaling, crusting, ulceration, or thickening. AK is a premalignant keratinocytic lesion associated with chronic ultraviolet exposure and may develop into SCC.

BKL represents benign keratotic lesions that can contain pigmentation, keratin structures, or irregular borders and may resemble malignant lesions. DF is a benign fibrohistiocytic lesion that may show a scar-like center and peripheral pigment network. Vascular lesions, including angiomas and angiokeratomas, may appear red, purple, blue, or dark and can occasionally resemble pigmented malignancies. These similarities emphasize the need for diverse datasets and robust validation when developing automated classification systems. Figure 2 provides representative dermoscopic examples of the major lesion categories discussed in this section.

**Figure 2:**
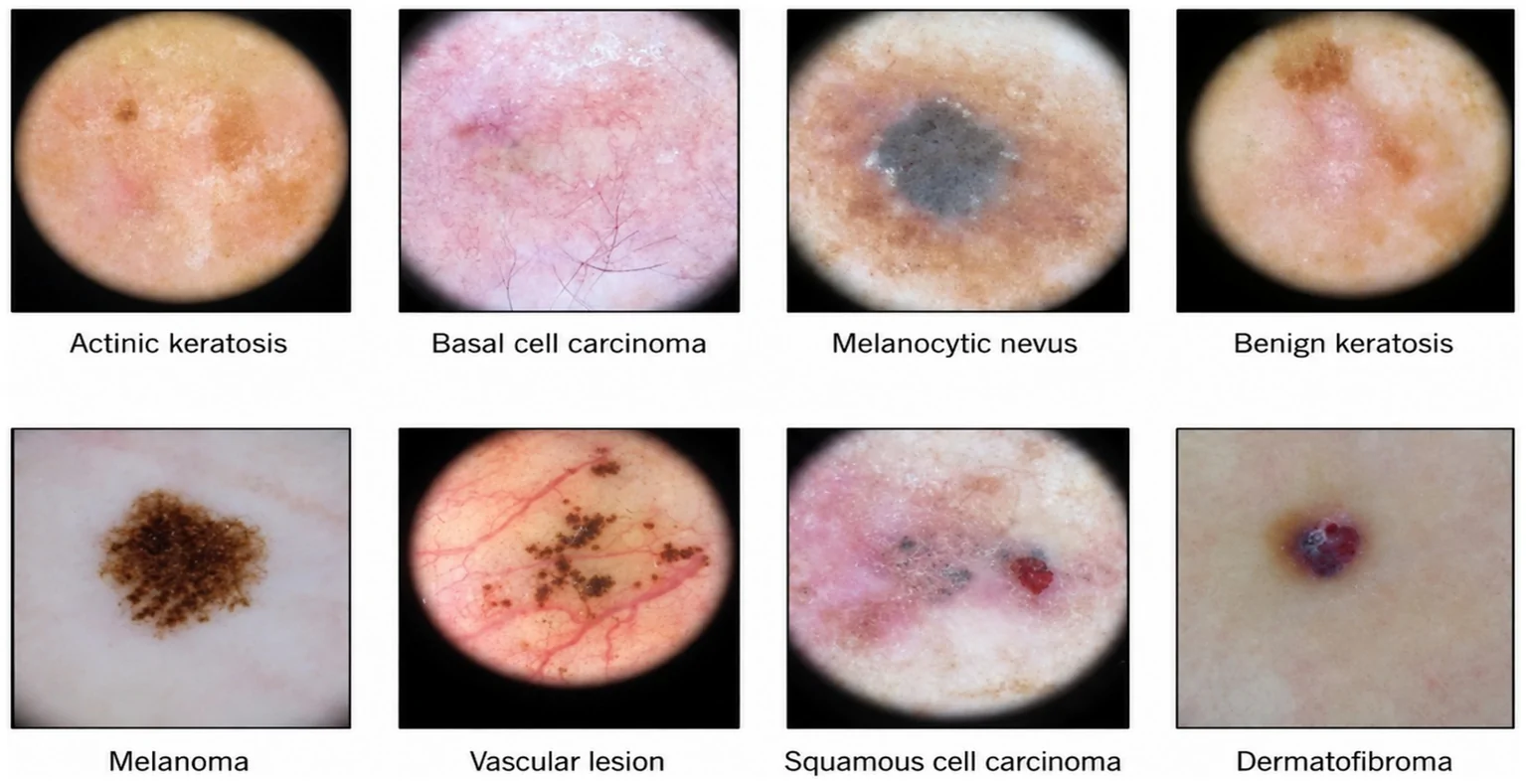
Representative dermoscopic images of Actinic Keratosis (AK), Basal Cell Carcinoma (BCC), Benign Keratosis (BKL), Dermatofibroma (DF), Melanoma (MEL), Melanocytic Nevus (NV), Squamous Cell Carcinoma (SCC), and Vascular Lesion (VASC) from the International Skin Imaging Collaboration dataset.

### 2.2 Skin imaging datasets

Available dermoscopic imaging datasets have served as a foundation in creating and testing of automated skin lesion analysis algorithms. They include annotated images for segmentation, classification, lesion-attributes detection, model comparison, and reproducibility. Nonetheless, these datasets vary in image source, acquisition method, class distribution, diagnosis validation, annotations, patient population, and analytical task.

#### 2.2.1 HAM10000

The HAM10000 dataset consists of 10,015 multi-source dermoscopic images of seven common types of pigmented skin lesions including melanoma, melanocytic nevus, basal cell carcinoma, actinic keratosis and intraepithelial carcinoma, benign keratosis, dermatofibroma, and vascular lesions. The diagnoses have been validated via histopathological assessment, consensus, follow-up, or confocal microscopy (Tschandl et al., 2018). HAM10000 is popular for multiclass classification since it offers a sufficiently large number of dermoscopic images. Still, the dataset is highly imbalanced and includes a majority class of melanocytic nevi and several categories that include much fewer images clinically. Therefore, overall accuracy alone might not reflect a sufficient performance of the algorithm. The metrics to consider include balanced accuracy, sensitivity, specificity, precision, F1-score, confusion matrix, and Area Under the ROC Curve.

#### 2.2.2 PH^2^ dataset

PH^2^ dataset includes dermoscopic images of typical melanocytic lesions, such as benign and atypical nevi and melanoma. The dataset includes expert annotations and manual delineation of lesions. Because of its small size, in comparison to other ISIC datasets, the PH^2^ dataset is often used for external validation.

#### 2.2.3 Derm7pt dataset

Derm7pt dataset includes both dermoscopic and clinical images with structured information according to the seven-point dermoscopic checklist. The combination of images, diagnostic annotations, and clinical information is very useful for multimodal system evaluation. However, one should consider the problems of incomplete metadata and the differences in dermoscopic and clinical images capturing.

#### 2.2.4 International skin imaging collaboration archive

The International Skin Imaging Collaboration Archive is a large archive of images that is being constantly extended and serves as a source of data for various challenges aimed at analyzing skin lesions. The ISIC challenge versions include challenges for tasks such as lesion segmentation, lesion-attribute detection, multiclass classification, and analysis based on clinical metadata.

ISIC data archives served as a basis for comparison of various segmentation and classification algorithms. Nevertheless, the individual ISIC challenge archives differ from each other in terms of size, distribution of classes, type of images, annotation procedure, and evaluation protocol. Therefore, results obtained with ISIC-2016, ISIC-2017, ISIC-2018, and ISIC-2019 cannot be considered equivalent without taking these differences into account.

ISIC images include various realistic changes that occur in the images captured in the clinical environment (e.g., variation in illumination conditions, magnification, size of the lesion, hair occlusion, rulers, color charts, bubbles, and image borders).

#### 2.2.5 Considerations related to the datasets

Publicly available datasets have facilitated the model development process but excellent results on one specific benchmark do not guarantee generalization to the practice. Key dataset-related factors to consider are as follows:

1. Class imbalance between common benign lesions and rare malignant classes.
2. Lack of diversity in dark-skinned individuals and other minorities.
3. Illustrations obtained with different tools and lighting.
4. The presence of several images per patient or lesion.
5. Inconsistency in diagnosis confirmation and annotations.
6. Differences between dermoscopic images and regular clinical pictures.
7. Shortcut learning through rulers, markings, borders and other artifacts.

Patient separation is required where patient IDs are available to avoid using related images for both training and testing. External validation through an independent dataset is a stronger indication of generalization than random splitting of one dataset.

### 2.3 Relevance of image analysis

Most approaches for automated skin lesion analysis usually rely on two interrelated operations:

1. Segmentation, which entails detecting the region of the lesion and separating it from the rest of the skin.
2. Classification, which implies categorization of the whole image or segment of the lesion.

The advantage of segmentation is that it allows reducing the background noise and extracting color, texture, shape, and border characteristics of the lesion. The disadvantage of segmentation lies in the possible misclassification and omission of clinically relevant information or excess of healthy skin, which will affect further classification of the image. Some integrated approaches try to optimize the process of segmentation and classification together or base classification on the results of segmentation (Al-Masni et al., 2020).

Conventional machine learning models are usually based on manual feature engineering, including color histograms, texture attributes, border irregularity, asymmetry, and geometric characteristics. Models with CNNs are capable of learning hierarchical representations from images and are extensively employed for skin lesion classification and segmentation. The U-Net architecture along with other encoder-decoder CNNs is usually utilized for pixel-wise localization of lesions, while ResNet, DenseNet, EfficientNet, MobileNet, and many other CNN models are commonly applied for classification tasks. Hybrid approaches integrate segmentation and classification tasks, handcrafted and deep features, or CNN and Transformer representations to leverage complementary information (Shetty et al., 2022; Benyahia et al., 2022).

The performance of any automated method cannot be assessed solely by the best accuracy obtained by the system. Such aspects as dataset composition, class balancing, patient splitting, computational constraints, robustness, interpretability, and external validation are also worth considering. Table 2 presents representative CNN-based, hybrid, self-supervised, and Transformer-based approaches used for skin lesion analysis.

CNN-based techniques are still popular since they have the ability to learn specific lesion color, texture, border, and structure features directly from images. Segmentation–classification techniques aim at minimizing the effect of the background by detecting the lesion area first, while transfer learning and lightweight approaches try to minimize training needs or facilitate implementation with limited resources.

It should be noted that the table does not represent an exact ranking of models since the studies employed in it differ in terms of data used, lesions, resolutions, preprocessing, data splits, architectures, and metrics used. Hence, any difference in accuracy or segmentation results cannot be attributed solely to the architecture. Studies reporting external validation, independent data separation, and computational requirements provide additional evidence for assessing generalizability and deployment feasibility, however, these characteristics should still be interpreted in the context of the specific study design and dataset.

## 3 Image processing and preprocessing techniques

Image preprocessing is an important component of automated skin lesion analysis because dermoscopic images may contain hair, rulers, bubbles, shadows, illumination variation, sensor noise, color differences, and inconsistent image resolution. These factors can interfere with lesion-boundary detection, modify diagnostically relevant features, and cause models to learn acquisition-related artifacts rather than lesion characteristics.

The objective of preprocessing is therefore to reduce unwanted image variation while preserving clinically relevant information. Common operations include contrast enhancement, color normalization, hair and artifact removal, noise filtering, resizing, intensity normalization, and data augmentation. However, preprocessing should not be treated as a fixed sequence applied to every dataset. Its suitability depends on image characteristics, acquisition conditions, lesion properties, downstream task, and model architecture (Khattar and Kaur, 2022; Garcea et al., 2023).

### 3.1 Image enhancement and artifact correction

Image enhancement aims to improve the distinction between lesions and surrounding skin and to facilitate subsequent segmentation or classification. Common approaches include histogram equalization, Contrast-Limited Adaptive Histogram Equalization (CLAHE), contrast stretching, color-space transformation, color normalization, and artifact removal.

#### 3.1.1 Histogram equalization and CLAHE

Histogram equalization redistributes pixel intensities to increase global contrast and can be useful when intensity variation is limited. However, excessive enhancement may amplify noise and acquisition artifacts or alter lesion color distributions. Contrast-Limited Adaptive Histogram Equalization (CLAHE) performs localized enhancement while limiting excessive amplification and may be useful when illumination varies spatially or lesion boundaries have low local contrast. However, it can also enhance hair, bubbles, rulers, skin texture, and compression artifacts. Therefore, enhancement should be selected according to image characteristics and evaluated using downstream segmentation or classification performance rather than visual appearance alone.

#### 3.1.2 Contrast stretching and color-space transformation

Contrast stretching expands the intensity range and may improve lesion–skin separation when the available range is narrow, although its benefit can be limited by extreme intensity values or spatially varying contrast. Dermoscopic images are commonly represented in RGB, while HSV, CIELAB, and YCbCr provide alternative representations of color and intensity that may facilitate pigmentation, brightness, and texture analysis. No single color space is universally optimal, effectiveness depends on lesion characteristics, acquisition conditions, dataset composition, and model architecture. Deep networks may also learn useful color representations directly when training data are sufficiently diverse.

#### 3.1.3 Color normalization

Color normalization aims to reduce variability caused by illumination, cameras, dermoscope calibration, and institutional acquisition protocols. It can improve consistency across datasets and institutions but may also suppress clinically meaningful pigmentation patterns. Because color is diagnostically relevant, normalization should preserve relative color relationships within lesions and its parameters should be validated using downstream performance, particularly in cross-dataset and multi-institutional studies.

#### 3.1.4 Hair and artifact removal

Hair can interrupt lesion boundaries and interfere with feature extraction. Common approaches include morphological filtering, directional filtering, thresholding, DullRazor-like methods, and image inpainting. Bansal and Sridhar (2024) investigated GAN-based inpainting for hair-obscured skin lesion images. Artifact removal should be applied cautiously because vessels, pigment networks, streaks, and irregular lesion structures may resemble hair, while inpainting may introduce artificial texture or color. Other artifacts, including rulers, ink markings, color charts, bubbles, reflections, and dark borders, may require cropping, masking, morphological operations, or inpainting.

### 3.2 Noise reduction

Noise may arise from sensors, acquisition conditions, compression, illumination, or image transmission. Denoising can improve image consistency, but excessive filtering may remove diagnostically relevant boundaries, vessels, pigment networks, or fine texture.

Gaussian filtering performs local weighted averaging and can reduce approximately Gaussian noise but may blur lesion boundaries when applied excessively. Median filtering is useful for impulse or salt-and-pepper noise and generally preserves edges better than simple averaging, although large kernels may remove small structures. Anisotropic diffusion reduces noise while limiting smoothing across strong gradients, but its performance depends on parameters such as the number of iterations and diffusion coefficient.

Image-quality measures such as Peak Signal-to-Noise Ratio and Structural Similarity Index may be reported for denoising, but they do not establish whether clinically relevant information has been preserved. Therefore, denoising should ultimately be evaluated through downstream segmentation, classification, boundary, or class-specific performance and, where appropriate, expert assessment.

### 3.3 Data augmentation

Medical imaging datasets often contain limited labeled examples and substantial class imbalance. Data augmentation increases training diversity and can reduce overfitting and improve robustness to acquisition variation (Garcea et al., 2023). However, augmentation should remain clinically plausible and should not alter lesion characteristics in unrealistic ways.

#### 3.3.1 Geometric transformation

Common transformations include rotation, horizontal and vertical flipping, translation, cropping, scaling, zooming, and elastic or affine transformations. Rotation and flipping are often suitable because lesion location and orientation are not fixed. Cropping and scaling can improve scale robustness, whereas excessive cropping may remove lesion regions and extreme deformation may generate anatomically implausible examples.

For segmentation, every geometric transformation applied to an input image must also be applied consistently to its corresponding ground-truth mask.

#### 3.3.2 Photometric augmentation

Photometric augmentation modifies brightness, contrast, saturation, hue, sharpness, or color balance and can simulate acquisition variability. However, pigmentation and color patterns are diagnostically relevant, so excessive photometric transformation may change the perceived lesion characteristics. Such transformations should therefore remain within clinically realistic ranges.

#### 3.3.3 MixUp and CutMix

MixUp combines images and their labels, whereas CutMix replaces a region of one image with a region from another and updates the corresponding label. These methods can regularize models and reduce memorization (Lewy and Mańdziuk, 2023).

However, mixed images may contain color or texture combinations that do not correspond to realistic clinical lesions. Their effectiveness should therefore be evaluated using independent real-world images rather than augmented validation data alone.

#### 3.3.4 AutoAugment and RandAugment

AutoAugment searches for augmentation policies and magnitudes based on validation performance, whereas RandAugment applies a controlled set of random operations. These approaches can reduce dependence on manually designed policies but may increase computational requirements or generate clinically inappropriate transformations.

Selected augmentation policies should therefore be checked for their influence on lesion morphology, diagnostic features, and explanation stability (Liu et al., 2025).

#### 3.3.5 GAN- and diffusion-based augmentation

Generative Adversarial Networks can generate synthetic lesion images or reconstruct artifact-affected regions and may increase representation of underrepresented classes. However, GANs may suffer from mode collapse, memorization, limited diversity, or unrealistic structures.

Diffusion models provide another approach to synthetic image generation but require substantial computational resources. Alimisis et al. (2025) highlighted their potential for medical image augmentation while emphasizing the need for appropriate evaluation.

Synthetic images should complement rather than replace real clinical images. Their evaluation should consider expert assessment, similarity, diversity, minority-class representation, privacy and memorization, artifact generation, independent real-image performance, and subgroup behavior.

### 3.4 Normalization and resizing

The selected normalization should be compatible with the architecture and, where applicable, the Pre-training procedure.

#### 3.4.1 Normalization strategies

Normalization places image values within a consistent numerical range and can improve optimization stability. Min-max normalization commonly scales values to 0–1 and is simple but may be sensitive to outliers. Z-score normalization centers and scales values using the mean and standard deviation, which may be calculated per image, per channel, or from the training set, the selected strategy should therefore be reported explicitly. Pre-trained models require normalization compatible with their Pre-training procedure, such as the channel statistics used during ImageNet Pre-training. Input normalization should be distinguished from batch normalization, which operates on internal feature representations. For small batches, group, instance, or layer normalization may provide alternatives.

#### 3.4.2 Resizing images

Deep learning architectures generally require consistent input dimensions. Resizing should preserve lesion geometry because direct conversion of different aspect ratios to a square can deform lesion shape. Aspect-ratio-preserving resizing, padding, or appropriate cropping can reduce distortion, although padding may introduce artificial borders that become unintended model cues.

### 3.5 Practical pipeline for preprocessing

Preprocessing should be selected according to the dataset and task rather than by combining every available operation into a single pipeline.

#### 3.5.1 Pipeline a: standard transfer-learning classification

A classification pipeline may include:

1. Removal of invalid borders or regions,
2. Aspect-ratio-preserving resizing,
3. Moderate geometric and photometric augmentation,
4. Normalization compatible with the Pre-trained model, and
5. Color normalization when required for images from different sources.

This approach is applicable to commonly used CNN architectures such as ResNet, DenseNet, EfficientNet, and MobileNet.

#### 3.5.2 Pipeline B: boundary-oriented lesion segmentation

A segmentation-oriented pipeline may include:

1. Hair removal when necessary,
2. Contrast enhancement when lesion boundaries have low visibility,
3. Edge-preserving denoising,
4. Resizing with the same transformation applied to the ground-truth mask,
5. Pixel-value normalization, and
6. Geometric augmentation applied consistently to image and mask.

Such preprocessing can support U-Net, Attention U-Net, ResUNet, and related encoder–decoder architectures.

#### 3.5.3 Pipeline C: transformer-based analysis

Transformer-based analysis requires particular attention to consistent image or patch resolution, normalization compatible with Pre-training, clinically realistic augmentation, and preservation of intrinsic color and texture statistics. Excessive local contrast or color manipulation may change patch-level distributions and reduce transferability.

#### 3.5.4 Pipeline D: cross-dataset or multi-institutional analysis

Cross-dataset studies may require:

1. Device- and institution-aware color normalization,
2. Border and artifact detection,
3. Consistent resizing,
4. Dataset-aware augmentation,
5. External validation, and
6. Subgroup analysis across devices, populations, and skin-tone categories.

None of these pipelines should be selected merely to produce visually enhanced images. Their value should be demonstrated through segmentation or classification performance, robustness, and generalization. Table 3 summarizes representative preprocessing and augmentation studies, their contributions, and important limitations.

### 3.6 Critical comparison of preprocessing strategies

Preprocessing techniques provide different benefits and risks. Global histogram equalization is simple and useful for relatively uniform low contrast, whereas CLAHE can better address local illumination variation but may amplify artifacts. Color normalization can reduce acquisition-related variation but may also suppress diagnostically meaningful pigmentation. Hair removal can improve boundary continuity but may remove vessels, streaks, or pigment networks when incorrectly applied.

The central requirement is therefore preservation of diagnostically relevant information. Filtering and artifact correction should preserve lesion borders, pigment networks, streaks, dots, globules, vascular structures, regressed regions, and color asymmetry. An operation that produces a smoother or visually improved image but reduces downstream diagnostic performance should not be considered an improvement.

The appropriate preprocessing also depends on the architecture. CNNs learn local edges, textures, and color gradients and may benefit from moderate enhancement and augmentation, whereas excessive smoothing can remove features needed for learning. U-Net-based models may benefit from improved lesion–skin contrast and boundary continuity, but artificial edges introduced by aggressive enhancement or inpainting can produce incorrect segmentation masks. Vision Transformers rely on patch-level representations and therefore require consistent resolution and normalization, excessive color or contrast manipulation may alter patch statistics.

Augmentation should similarly be considered a modeling decision rather than a universal solution to class imbalance. Geometric transformations can improve invariance, whereas photometric transformations and synthetic generation must preserve clinically plausible lesion characteristics. Generative augmentation may increase representation of rare categories, but synthetic data require independent validation because visual realism does not establish diagnostic validity.

Overall, preprocessing and augmentation methods should not be considered universally beneficial. Their effectiveness depends on image characteristics, lesion appearance, acquisition conditions, dataset composition, and the downstream task. Garcia-Arroyo and Garcia-Zapirain (2019) illustrate the sensitivity of conventional segmentation to intensity, color, and illumination variation, whereas Ashraf et al. (2022) demonstrate how test-time augmentation and conditional random field refinement can improve mask consistency at the cost of additional inference complexity. Mahbod et al. (2019) further illustrates that multi-scale representation can improve the use of complementary image features but may increase computational and storage requirements. These studies indicate that preprocessing and augmentation should be selected according to the target dataset and clinical application rather than applied solely to improve benchmark performance. The representative studies and their methodological trade-offs are summarized in Table 3.

## 4 Segmentation techniques

Skin lesion segmentation separates the lesion from surrounding skin, reducing background interference and supporting subsequent feature extraction, classification, and measurement of characteristics such as size, asymmetry, and border irregularity. It is challenging because lesions may have irregular or poorly defined boundaries, heterogeneous pigmentation, low contrast, and varying sizes, while hair, rulers, bubbles, reflections, image borders, and illumination differences can further interfere with segmentation.

Segmentation approaches can be broadly grouped into image-processing methods, CNN-based methods, encoder–decoder and U-Net architectures, attention and multi-scale models, Transformer and CNN–Transformer approaches, and promptable medical foundation models.

### 4.1 Traditional segmentation methods

Traditional approaches use manually defined criteria based on intensity, color, texture, edges, or regions. They are generally computationally inexpensive and relatively interpretable, but their performance depends strongly on image quality, preprocessing, parameter selection, and lesion–skin contrast.

#### 4.1.1 Thresholding

Thresholding separates lesion and background according to pixel intensity or color. Global methods use a fixed threshold, whereas adaptive methods determine thresholds locally, Otsu’s method selects a threshold by maximizing separation between intensity distributions. Garcia-Arroyo and Garcia-Zapirain (2019) combined histogram thresholding with fuzzy pixel classification for dermoscopic lesion segmentation. Thresholding is simple and fast when lesion and surrounding skin have distinct characteristics, but performance decreases with heterogeneous pigmentation, non-uniform illumination, and poorly defined boundaries. Parameters selected for one dataset or acquisition system may therefore have limited transferability.

#### 4.1.2 Edge-based segmentation

Edge-based methods identify intensity or color transitions using operators such as Sobel, Prewitt, Canny, Laplacian, and gradient filters. They can provide fast initial contours when lesion boundaries are distinct, but dermoscopic boundaries are often weak or interrupted by hair, vessels, rulers, and image borders, resulting in inaccurate or spurious contours.

#### 4.1.3 Region-based segmentation

Region-growing methods expand from seed pixels according to intensity, color, or texture similarity, while watershed methods treat the image as a topographic surface. These approaches can produce connected lesion regions when boundaries are incomplete, but region growing depends on seed selection and watershed methods may suffer from over-segmentation in noisy images. Preprocessing and post-processing are often required to merge regions and remove isolated components.

#### 4.1.4 Clustering methods

Clustering groups pixels according to feature similarity without requiring pixel-wise labels. K-means assigns each pixel to a single cluster, whereas fuzzy C-means allows partial membership and can represent ambiguous lesion boundaries. Nida et al. (2019) combined deep region-based localization with Fuzzy C-Means for melanoma segmentation. However, clustering remains sensitive to initialization, cluster number, noise, illumination, and overlapping lesion–skin distributions.

#### 4.1.5 Active contours and energy-based methods

Active contour models evolve an initial contour through energy minimization using edge or region information. They can produce smooth boundaries for irregular lesions, but performance depends on initialization, energy formulation, iteration number, and parameter selection. Low gradients and heterogeneous lesion interiors may cause premature termination or leakage into surrounding tissue. Graph-cut methods similarly formulate segmentation as an energy-minimization problem in which pixel relationships are represented using graph structures.

#### 4.1.6 Critical assessment of traditional methods

Conventional methods are useful when computational capacity is limited, image acquisition is controlled, lesion–skin contrast is high, or transparent processing criteria are required. They can also serve as initial lesion-localization steps before applying more complex methods. However, their main limitation is reliance on manually defined assumptions. A threshold, color space, edge criterion, or clustering strategy that performs well under one set of conditions may not transfer reliably to another dataset or acquisition environment.

### 4.2 Feature extraction and pre-segmentation enhancement

Before deep learning became dominant, segmentation pipelines commonly used manually designed color, texture, shape, and edge features. Color features were derived from RGB, HSV, CIELAB, and related color spaces, while texture was represented using descriptors such as Gray-Level Co-occurrence Matrix (GLCM), Local Binary Patterns, wavelets, and statistical measures. Shape descriptors included lesion size, compactness, eccentricity, asymmetry, diameter, and border curvature.

Pre-segmentation enhancement may include contrast adjustment, hair and ruler removal, color normalization, and noise reduction. These operations can improve lesion–background separation but may also modify diagnostically relevant color, texture, or boundary information. Their selection should therefore be consistent with the segmentation task and validated using downstream performance.

### 4.3 Deep-learning-based segmentation

Deep learning techniques automatically acquire hierarchical lesion representations from the training images along with their corresponding masks. This decreases reliance on threshold selection and hand-crafted feature sets. Segmentation models based on deep learning can learn local edge, texture, color information, and lesion shape and context.

The basic need for such models is representative training data along with the availability of accurate ground truth masks. The models which have been trained based on unreliable or inaccurate annotations might learn to detect unstable boundaries of the lesions.

#### 4.3.1 Convolutional and fully convolutional networks

Learning features from images is done by way of convolutional filters in CNNs. Early CNN-based segmentation approaches adopted sliding windows or patch-based classification approach to decide whether a region in an image belonged to the lesion or not. Computationally intensive, these techniques repeatedly processed overlapping patches.

Fully Convolutional Networks utilized convolution operations instead of fully-connected layers and made pixel-level prediction on whole images. This breakthrough paved the way for efficient end-to-end semantic segmentation tasks. In their study, Al-Masni et al. (2018) utilized deep full-resolution convolutional network for dermoscopic lesion segmentation and proved the importance of maintaining spatial information when making pixel-level predictions.

CNNs are powerful tools in learning local edges, textures, and color gradients. Nonetheless, repeated downsampling may wash away fine details on boundaries, while the local receptive field in the beginning makes representation of the global shape of lesion difficult. The general stages of feature learning and prediction in CNN are depicted in Figure 3.

**Figure 3:**
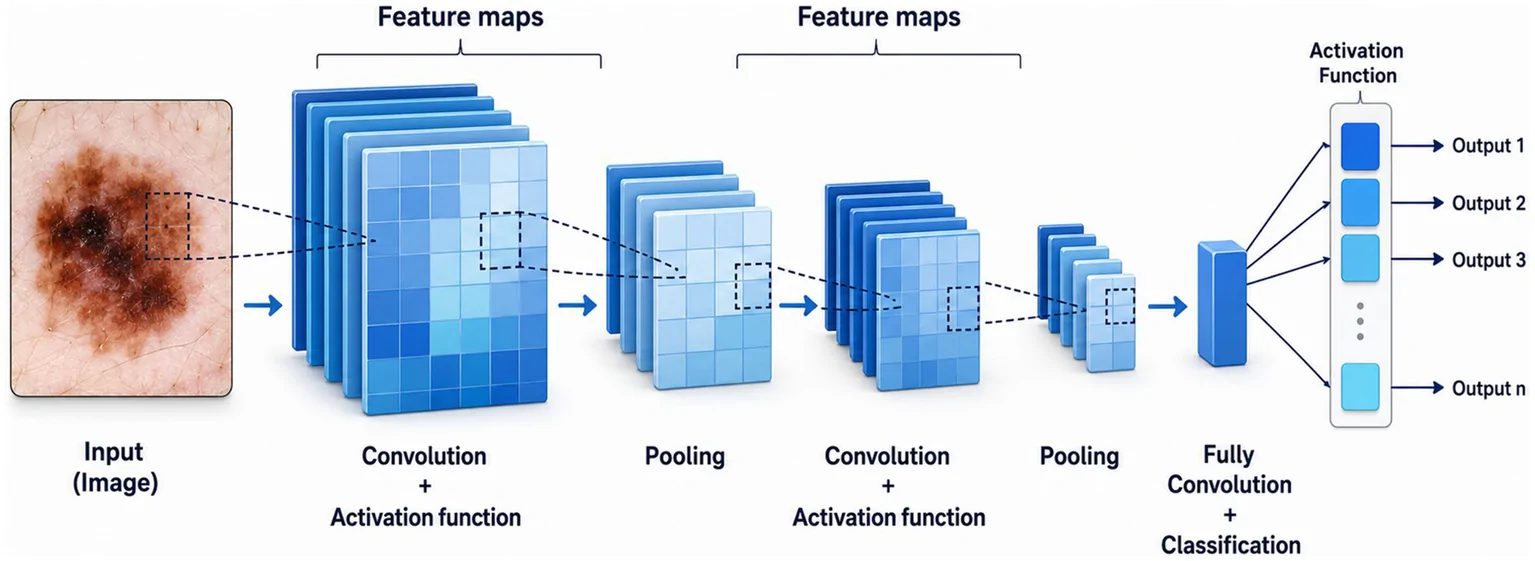
General workflow of a convolutional neural network showing convolution, activation, pooling, hierarchical feature extraction, and prediction stages.

#### 4.3.2 U-net and its variants

U-Net is an encoder-decoder neural network designed for medical image segmentation. The encoder decreases spatial resolution but extracts semantic features. The decoder increases the spatial resolution and produces pixel-wise segmentation masks. Skip connections allow transferring high-resolution features from the encoder part to the corresponding decoder layers.

Both semantic and spatial information contribute to U-Net’s ability to work successfully with not much annotated data. Nonetheless, conventional U-Net can face the problem of semantic mismatch between low-level features of the encoder and high-level features of the decoder. Downsampling can result in poor representation of small lesions and fine boundaries.

Different variations of U-Net are available:

- Residual U-Net: Adds residual connections to increase gradient propagation and allows deep training.
- Attention U-Net: Employs attention gates to ignore background features and highlight the lesion region.
- U-Net++: Designs nested and redesigned skip connections to minimize the semantic mismatch between encoder and decoder features.
- MultiResUNet: Uses multi-resolution convolutional blocks to encode structures on various spatial scales (Ibtehaz and Rahman, 2020).
- TransUNet: Unifies convolutional feature extraction, global-context modeling with Transformer architecture, and decoding similar to U-Net (Chen et al., 2024).

The U-Net modifications usually offer good boundary localization, but the inclusion of residual blocks, dense blocks, attention mechanisms, or Transformers adds to the complexity of the network. This needs to be proven using ablation studies carried out under the same experimental conditions. Figure 4 summarizes the architecture of the encoder-decoder of the U-Net.

**Figure 4:**
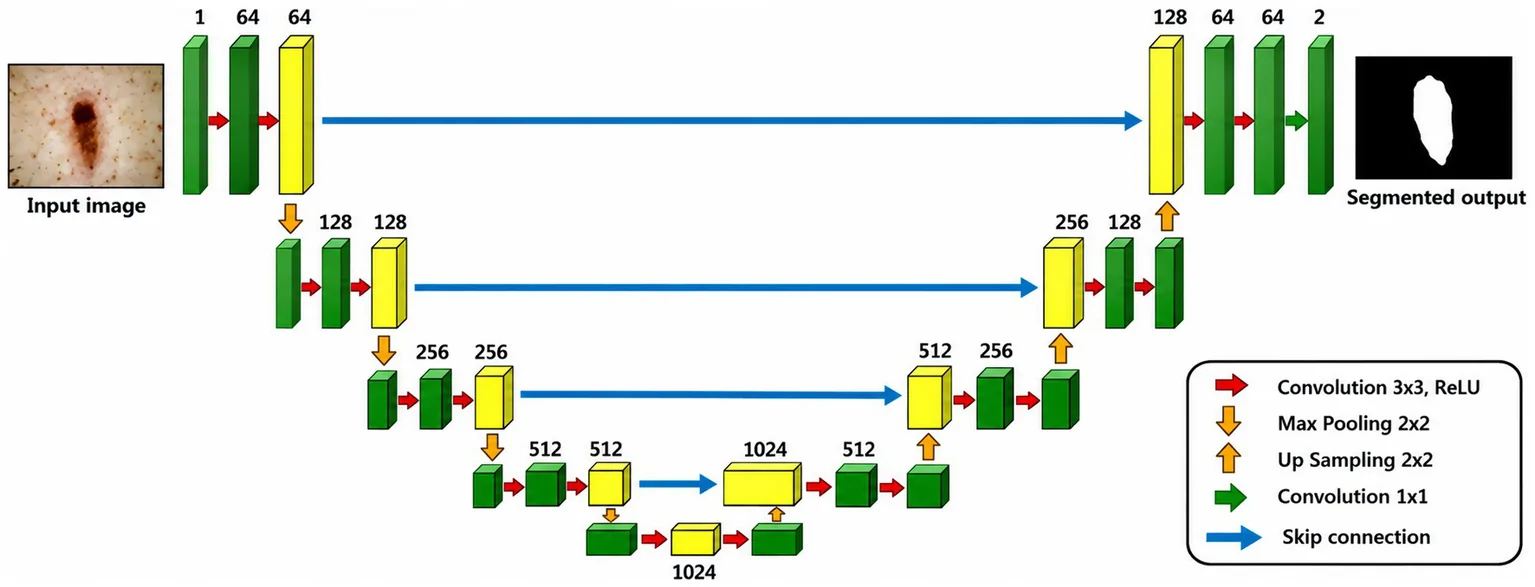
General U-Net architecture showing the encoder, bottleneck, decoder, skip connections, and pixel-level lesion mask.

#### 4.3.3 SegNet and lightweight encoder–decoder models

In SegNet, the encoder-decoder approach is used, whereby the decoder makes use of the pooling indices generated by the encoder. The pooling indices allow for a reduction in the parameters needed during upsampling.

Lightweight encoder-decoder models like SegNet could be used in time-critical applications. These models could have weaker boundary recovery compared to those with numerous skip connections and multiscale feature fusion.

The selection of either the lightweight model or the more complex model will depend on several factors including inference latency, memory usage, energy efficiency, segmentation accuracy, and clinical significance of fine boundaries.

#### 4.3.4 Residual and multi-resolution networks

Identity connections are included in residual segmentation models to ensure feature data and gradients pass through convolutional layers without being obstructed. This helps avoid optimization issues in deep networks. ResUNet is based on residual connections combined with the U-Net encoder-decoder architecture.

Multi-resolution approaches handle features using multiple resolutions. Large receptive field sizes help determine the shape of the whole lesion, whereas small receptive fields help to preserve texture and boundary information. It is helpful due to a large variation in skin lesion sizes.

High-resolution approaches keep feature maps at relatively high resolution, and exchange information between the scales repeatedly. Xie et al. (2020) implemented a high-resolution CNN to perform skin lesion segmentation, showing the need to keep spatial information.

These types of networks usually offer better representation, although they also need more memory and computation power. The complexity of the models has to be justified by a significant increase in performance.

#### 4.3.5 Attention-based segmentation

Attention mechanisms give higher relevance to pertinent areas within an image or feature channels. Spatial attention gives relevance to lesion areas, whereas channel attention gives importance to the discriminative feature maps. Attention mechanisms are also capable of suppressing the responses from background areas via skip connections.

The importance of attention mechanisms was shown by Schlemper et al. (2019) for medical segmentation models to pay attention to relevant areas. For the task of skin lesion segmentation, attention mechanisms will help to ignore the influence of normal skin, hair, rulers, and other background elements. A study by Alahmadi (2022) studied a multi-scale Attention U-Net model for skin lesion segmentation.

Attention does not mean that clinically relevant areas are focused upon necessarily. Attention maps can still include artifacts or information specific to the datasets. The importance of attention mechanisms should be confirmed using ablation studies and visualization.

#### 4.3.6 Auxiliary-task and boundary-aware segmentation

Auxiliary tasks add extra supervision to training. Segmentation models can learn lesion boundaries, distance maps, lesion classification, image reconstruction, or lesion shape along with other things. Liu et al. (2021) utilized an auxiliary task for better skin lesion segmentation. Boundary-aware approaches directly penalize for differences at lesion boundaries or fuse regional and boundary segmentations. This kind of approach is very beneficial when the lesion interior is easy to detect while the boundary is not clear. The use of auxiliary supervision helps with learning features but might bring optimization challenges due to the addition of new tasks.

#### 4.3.7 Post-processing

The predicted masks could also have isolated false positives, holes, irregular boundaries, or even disconnected components. The post-processing approaches are as follows:

1. Morphological opening and closing,
2. Filling of holes,
3. Removing of small connected components,
4. Keeping of the largest connected component,
5. Conditional Random Fields, and
6. Smoothing of contours.

Ashraf et al. (2022) used the combination of deep segmentation, test-time augmentation, and Conditional Random Fields refinement for melanoma segmentation. Even though the post-processing process could increase the regularity of the mask, it could hide possible deficiencies in the network itself. It means that the model needs to be tested both before and after the post-processing process.

### 4.4 Transformer-based segmentation

Transformers learn the interaction between spatially distant parts of an image through the self-attention mechanism. Such a global interaction could be beneficial for skin lesions as clinically significant structures might be located in different parts of the lesion and the lesion shape might help in their localization. As compared to traditional CNNs, Transformers do not have many intrinsic assumptions about locality and translation properties. They usually need more data, augmentation, large-scale pre-training, or even hybrid convolutional modules.

#### 4.4.1 TransUNet

TransUNet consists of convolutional local feature extraction along with Transformer-based global context modeling and U-Net-style decoding. The Transformer encoder learns the relationship between spatially distant features, whereas the decoding part helps recover high-resolution information through skip connections. In Chen et al. (2024), the journal version of TransUNet is introduced and the design of U-Net segmentation with Transformers is studied. TransUNet is helpful for irregular lesions but usually consumes more resources than a classical U-Net.

#### 4.4.2 Swin-UNet-related models

The Swin Transformer model applies hierarchical shifted window attention. In contrast to global unrestricted attention, the attention is calculated in local windows, and the windows are shifted between subsequent stages. It decreases computational complexity but allows interaction between windows.

Swin-UNet-related architectures apply such hierarchical representation together with encoder-decoder reconstruction. They can capture multi-scale context but will not be able to capture very small structures if the patch division and down-sampling are not appropriately designed. Yao et al. (2024) reviewed the evolution from CNNs to Transformer architecture for medical image segmentation and described such architectures as TransUNet, Swin-UNet, and SAM-related.

#### 4.4.3 SegFormer-type architectures

SegFormer-type architectures unite hierarchical Transformer encoders and lightweight decoders. The fusion of multi-scale Transformer features creates the segmentation mask. The lightweight decoders make it possible to decrease part of the computational burden of encoder-decoder architectures.

For skin lesion segmentation, the SegFormer-type architectures require domain-specific validation since the preservation of the fine borders depends on image resolution, patch representation, decoder architecture, and Pre-training. The comparison of SegFormer-type models with U-Net should be based on the same dataset and splits and pre-processing.

#### 4.4.4 Skin-lesion-specific transformer models

Recent models for skin lesions employ the Transformer-based architecture for lesions’ textures, structures, and multi-scale features. Xin et al. (2024) offered a Transformer guided self-adaptive framework for segmentation of multiscale skin lesions based on the positional encoding, multi-scale features, and cross-attention mechanism. Xu et al. (2024) introduced the SkinFormer model that utilizes the structural information and statistical representation of textures due to the Transformer-based feature fusion. Wang Z. et al. (2025) introduced the Superpixel Embedded Transformer where the authors used superpixels as an alternative to rectangular patches to create tokens.

### 4.5 Foundation models for skin lesion segmentation

Foundation models are Pre-trained on large and diverse datasets and can support multiple downstream tasks. The Segment Anything Model (SAM) introduced promptable segmentation using points, bounding boxes, and mask prompts. In skin lesion analysis, promptable models may support preliminary mask generation, physician-assisted correction, annotation, lesion initialization, and semi-automatic segmentation. However, natural-image Pre-training may produce inadequate masks for poorly defined or low-contrast lesions, hair occlusions, and uneven pigmentation. MedSAM extends promptable segmentation to medical image-mask data (Ma et al., 2024), but task-specific dermoscopic validation remains necessary. SAM- and MedSAM-based approaches should therefore be evaluated for prompt sensitivity, boundary accuracy, small-lesion performance, artifact robustness, skin-tone and device variability, computational demands, uncertainty, and generalizability. Foundation models should be considered complementary tools rather than automatic replacements for specialist skin lesion segmentation networks.

### 4.6 Critical comparison and model-selection guidance

Segmentation approaches differ in computational requirements, data dependence, robustness, boundary representation, and generalization. Classical thresholding, clustering, graph-based, and morphology-based methods require relatively limited computational resources and can be useful as low-resource baselines or under controlled acquisition conditions, but their dependence on manually selected parameters can reduce robustness to illumination changes, artifacts, and poorly defined lesion boundaries.

CNN-based and U-Net-based approaches provide learned feature extraction and effective spatial localization, while skip connections support recovery of fine lesion boundaries. They are generally practical when pixel-level annotations and moderate training resources are available. However, performance may remain sensitive to annotation quality, downsampling, dataset-specific artifacts, and cross-institutional domain differences. Advanced attention and multi-scale variants can improve contextual feature integration but may introduce additional parameters and computational requirements.

Transformer and CNN–Transformer models explicitly model long-range relationships and can integrate global and multi-scale information. Their practical use may require suitable Pre-training, sufficient data, and greater memory and computational capacity. Foundation models such as SAM and MedSAM provide promptable segmentation and may reduce annotation effort or support interactive workflows. Importantly, MedSAM was developed and evaluated using a large medical segmentation collection that included dermoscopic imaging, with internal and external validation. Therefore, the remaining concern for skin-lesion research is not the absence of dermoscopic data in MedSAM development, but the need for independent external validation on dermoscopic datasets that are distinct from those used during foundation-model development. The reviewed skin-lesion studies illustrate the importance of this distinction: DSNet (Hasan et al., 2020) was evaluated on ISIC-2017 and PH2, while SapFormer (Xin et al., 2024) was evaluated on ISIC-2016, ISIC-2017, and ISIC-2018. These cross-dataset evaluations provide stronger evidence of generalization than evaluation on a single internal split.

Model selection should be based on dataset size and diversity, annotation availability, lesion characteristics, hardware, inference requirements, robustness, interpretability, validation design, and intended clinical use. Reported performance from different studies should not be directly ranked when datasets, preprocessing, annotations, data splits, and evaluation protocols differ substantially. The studies summarized in Table 4 illustrate these differences across classical methods, CNN/U-Net architectures, attention and multi-scale models, Transformer-based approaches, and foundation-model methods. For example, Al-Masni et al. (2018) evaluated on ISBI 2017 and PH2, Hasan et al. (2020) evaluated DSNet on ISIC-2017 and PH2, and Xin et al. (2024) evaluated SapFormer on ISIC-2016, ISIC-2017, and ISIC-2018. These examples show that cross-dataset testing is present in some representative studies, whereas many other studies rely on internal benchmark splits. Accordingly, dataset identity and validation design should be considered together when interpreting reported segmentation performance.

The studies summarized in Table 4 illustrate these differences across classical methods, CNN/U-Net architectures, attention and multi-scale models, Transformer-based approaches, and foundation-model methods, with corresponding differences in annotation requirements, computational cost, robustness, and validation needs.

### 4.7 Skin lesion segmentation evaluation measures

No single metric can fully describe segmentation performance. Evaluation needs to include both region overlapping and boundary-based evaluation.

Let TP be the pixels of lesion predicted correctly, TN pixels of background predicted correctly, FP pixels of background mispredicted as lesions, and FN pixels of lesion mispredicted as background.

#### 4.7.1 Dice similarity coefficient

Dice Similarity Coefficient is an overlap measure between prediction and ground truth mask:

$$\mathrm{DC}=\frac{2\mathrm{TP}}{2\mathrm{TP}+\mathrm{FP}+\mathrm{FN}}$$

Dice varies from 0 to 1, with 1 signifying perfect overlap. Dice is most commonly utilized when the lesion covers a fairly small area on the image.

Dice is responsive to overlap but does not always account for any serious error at the border.

#### 4.7.2 Intersection over Union

Intersection over Union or Jaccard Index is described as:

$$\mathrm{IoU}=\frac{\mathrm{TP}}{\mathrm{TP}+\mathrm{FP}+\mathrm{FN}}$$

IoU heavily penalizes dissimilarities and always yields a lower score than Dice. It is appropriate for measuring the level of region overlap.

#### 4.7.3 Pixel accuracy

Pixel accuracy is computed by:

$$\mathrm{Pixel Accuracy}=\frac{\mathrm{TP}+\mathrm{TN}}{\mathrm{TP}+\mathrm{TN}+\mathrm{FP}+\mathrm{FN}}$$

Accuracy may give a false impression as healthy skin frequently covers much of the dermoscopic image, and a model can score high accuracy even when the lesion is poorly segmented as long as it identifies the background correctly.

#### 4.7.4 Hausdorff distance

Hausdorff Distance is defined as the maximum distance between any pair of points from the boundaries of the predicted and reference regions. It can effectively identify gross discrepancies in segmentation where region overlap metrics would fail.

Maximum Hausdorff Distance can be sensitive to isolated outliers and thus 95th percentile Hausdorff Distance is used more often.

#### 4.7.5 Average surface distance

Average Surface Distance is calculated as the average distance between the predicted and reference boundaries. It is a more stable metric compared to maximum Hausdorff Distance, yet may ignore a few severe outliers.

#### 4.7.6 Recommended combination of metrics

For segmentation evaluation, it is preferable to state:

1. Dice similarity coefficient or IoU for measuring overall region overlap,
2. Hausdorff distance or average surface distance for measuring the quality of boundaries,
3. Sensitivity for measuring missed lesion regions,
4. Precision for measuring falsely detected lesion regions,
5. Specificity if there is any background in the task, and
6. Model parameters such as size, computational operations, memory usage, and inference time if the claim is about real-time or mobile implementation.

Two models that have equal Dice can be very clinically different. While one can make an error at the boundaries of the complete lesion structure, another can completely fail to detect a small but clinically significant part of it.

## 5 Skin lesion classification methods

Skin lesion classification assigns dermoscopic or clinical images to diagnostic categories such as melanoma, melanocytic nevus, basal cell carcinoma, actinic keratosis, benign keratosis, dermatofibroma, squamous cell carcinoma, and vascular lesions. Classification may use the full image, a region of interest, or a lesion mask generated by segmentation. Its clinical roles include screening, triage, diagnostic support, and prioritization of suspicious cases, however, false-negative malignant predictions may delay treatment, whereas false positives may lead to unnecessary referrals or biopsies. Therefore, evaluation should consider class-wise sensitivity, specificity, calibration, uncertainty, and generalizability in addition to overall accuracy. Classification approaches can be broadly grouped into handcrafted-feature machine learning, CNN-based methods, transfer learning, attention and Transformer models, ensembles and hybrid systems, self-supervised and multimodal approaches, and explainable or uncertainty-aware systems. The main steps in automatic skin lesion classification are shown in Figure 5.

**Figure 5:**
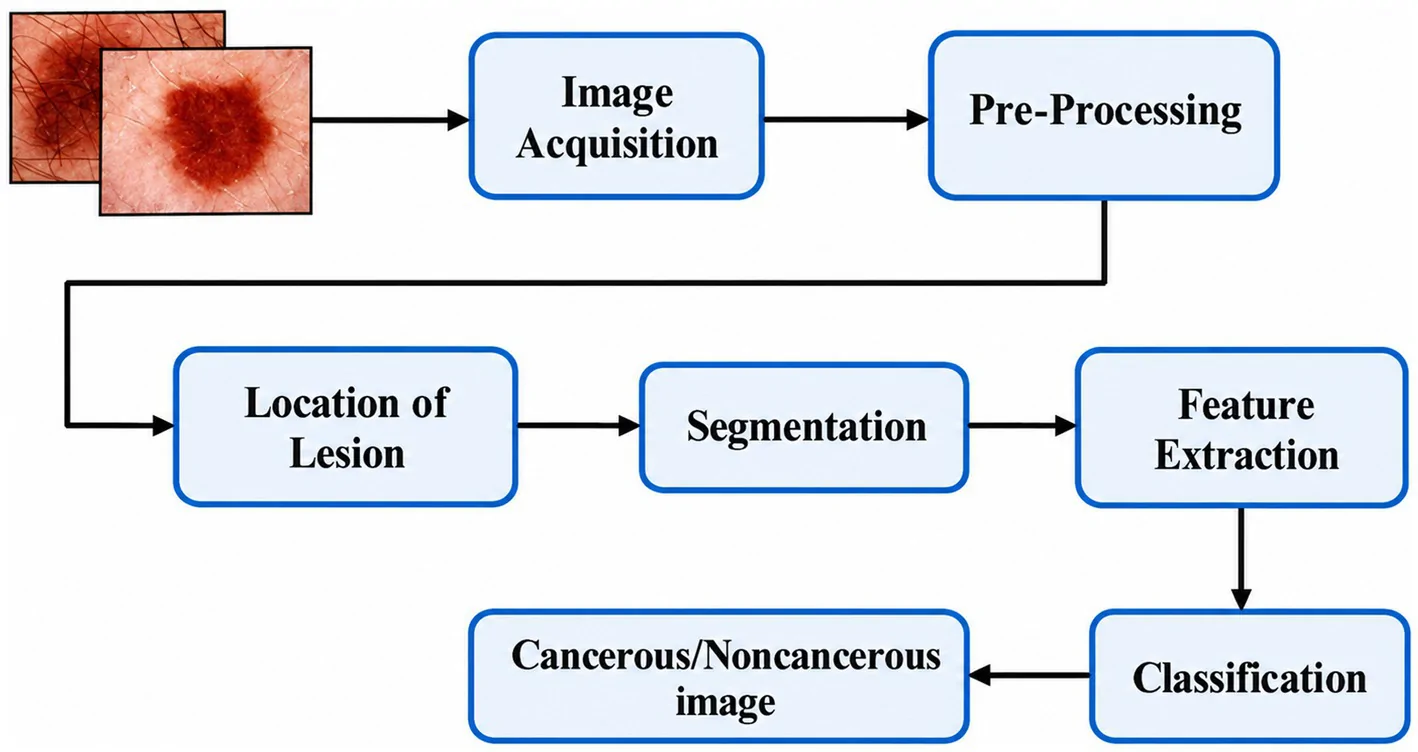
General workflow of an automated skin lesion classification system, including image acquisition, preprocessing, lesion localization, segmentation, feature extraction, and diagnostic classification.

### 5.1 Traditional machine-learning approaches

Traditional approaches classify lesions using manually designed color, shape, texture, and clinical descriptors followed by classifiers such as Support Vector Machines (SVM), K-Nearest Neighbors (KNN), Random Forests (RF), Decision Trees, Naïve Bayes, Logistic Regression, and shallow neural networks. They remain relevant for small datasets, limited computational resources, and applications requiring relatively interpretable feature-based pipelines.

Color descriptors may include RGB, HSV, or CIELAB statistics, histograms, color moments, and measures of pigmentation heterogeneity. Shape descriptors include area, perimeter, circularity, eccentricity, diameter, asymmetry, solidity, and border irregularity, although these features depend on accurate lesion masks. Texture descriptors include Gray-Level Co-occurrence Matrix features, Local Binary Patterns, Gabor filters, wavelets, entropy, contrast, correlation, and homogeneity. Clinically inspired descriptors may incorporate asymmetry, border irregularity, color, diameter, pigment networks, dots, globules, and vascular structures. Samsudin et al. (2022) demonstrated the use of multi-resolution empirical-mode decomposition and Local Binary Pattern features, while Almutairi and Khan (2023) examined classical image features and machine-learning methods for skin cancer analysis.

SVMs are effective when feature dimensionality is high relative to sample size, but their performance depends on kernel and regularization choices. KNN is simple and useful for small datasets but is sensitive to feature scaling, noise, distance measures, and the choice of k (Hatem, 2022). Decision Trees provide transparent decision rules but may overfit, whereas RF combines multiple trees and can model nonlinear relationships while reducing dependence on individual trees. Naïve Bayes is computationally efficient but assumes conditional feature independence, while Logistic Regression provides interpretable probabilistic coefficients but may be inadequate for highly nonlinear lesion patterns.

Overall, conventional methods offer relatively low computational requirements and can exploit clinically meaningful descriptors, but they depend strongly on segmentation quality and feature engineering. Their generalization may deteriorate when illumination, acquisition devices, skin tone, lesion appearance, or image quality changes. Thus, they remain useful under constrained conditions but are less suitable when large and heterogeneous image datasets permit end-to-end representation learning.

### 5.2 Classification based on deep learning

Deep learning methods learn hierarchical image representations directly from training data, reducing dependence on manually engineered features. CNNs learn local edges, color patterns, textures, and progressively higher-level lesion structures, while transfer learning enables Pre-trained networks to be adapted to dermoscopic datasets. Shetty et al. (2022) demonstrated the application of CNN-based classification to dermoscopic images, but high internal performance should be interpreted cautiously because models may learn dataset-specific shortcuts such as rulers, image borders, color charts, or acquisition-related patterns.

Common CNN backbones include VGG, ResNet, DenseNet, Inception, EfficientNet, and MobileNet. VGG architectures provide a simple sequential structure but contain substantial parameter counts. ResNet uses residual connections to facilitate deeper networks and has become a common transfer-learning backbone, although computational and overfitting concerns remain for small datasets. DenseNet promotes feature reuse through dense connectivity but can increase memory requirements. Inception uses parallel convolutional scales and can represent lesions with different spatial structures, although its architecture is more complex. EfficientNet balances network depth, width, and input resolution, while MobileNet uses depthwise separable convolutions to reduce computational requirements and is therefore relevant to mobile and point-of-care applications (Yilmaz et al., 2022).

Transfer learning can reduce training requirements by adapting representations learned from large datasets. Strategies include using a Pre-trained network as a feature extractor, replacing the classification head, fine-tuning selected layers, or progressively unfreezing deeper layers. Mahbod et al. (2019) used deep-feature fusion, while Mahbod et al. (2020) investigated multi-scale and multi-network transfer learning. Nevertheless, natural-image Pre-training may introduce domain mismatch because dermoscopic images differ from the source data. Fine-tuning should therefore use appropriate normalization, regularization, augmentation, learning-rate selection, and patient-independent data partitioning.

CNN-based approaches generally provide a practical balance between representation capacity and computational cost. However, increasing architectural depth or complexity does not necessarily improve external generalization. Controlled ablation, patient-level separation, external validation, and analysis of acquisition-related shortcuts remain important.

### 5.3 Attention, transformer, ensemble, and hybrid classification

Attention mechanisms increase the contribution of selected spatial regions or feature channels. Channel attention, spatial attention, squeeze-and-excitation blocks, convolutional block attention modules, attention gates, and self-attention have been used to emphasize diagnostically relevant lesion features. Qian et al. (2022) combined multi-scale attention with class-specific loss weighting, while Nguyen et al. (2022) investigated soft attention under class imbalance. However, attention maps should not automatically be interpreted as explanations because a model may attend to artifacts or other non-diagnostic regions. Component-wise ablation and explanation validation are therefore necessary.

Vision Transformers divide images into patches and use self-attention to model relationships between spatially distant regions. This global representation may capture asymmetry, diffuse pigmentation, and broader lesion structure. Cirrincione et al. (2023) examined Transformer-based melanoma classification (Khan et al. 2023). Compared with CNNs, Transformers generally depend more strongly on Pre-training, augmentation, computational resources, and adequate data, while patch-based processing may weaken local inductive bias. CNNs and Transformers should therefore be regarded as complementary approaches rather than universal substitutes.

Ensemble learning combines multiple models through majority voting, probability averaging, weighted averaging, stacking, or feature fusion. Popescu et al. (2022) investigated collective predictions from multiple neural networks, while Mahbod et al. (2020) and Shakourian Ghalejoogh et al. (2020) explored multi-network and stacking-based approaches. Ensembles may reduce dependence on individual models when their errors are complementary, but they increase training, storage, inference, and energy requirements.

Hybrid systems integrate different representations or tasks, including handcrafted and learned features, multiple CNN backbones, CNN-Transformer combinations, segmentation and classification, or image features with clinical metadata (Yang and Wang, 2023). Al-Masni et al. (2020) integrated lesion segmentation and classification, while Alam et al. (2022) proposed S2C-DeLeNet to transfer information between segmentation and classification. Such integration can focus classification on lesion regions, but segmentation errors may propagate to classification. Hybrid systems should therefore report the effect of segmentation errors, component-wise contributions, computational cost, and controlled ablation results. Table 5 summarizes the representative classification frameworks along with the limitations of each framework.

### 5.4 Critical comparison of classification approaches

Classification approaches differ in their dependence on labeled data, computational resources, representation learning, class distribution, interpretability, and validation design. The representative studies considered in Table 5 include conventional machine-learning and CNN-based methods, attention-based approaches, ensemble and hybrid systems, Transformer-based classification, self-supervised learning, and multimodal learning. These approaches illustrate different trade-offs between predictive performance, computational complexity, data requirements, and generalizability. Foundation-model approaches are discussed separately in Section 5.5 because their role extends beyond conventional supervised classification.

The evidence indicates that no single classification architecture is universally optimal. Traditional machine-learning methods remain appropriate when datasets are small, computational resources are limited, or feature-level interpretation is important. CNNs provide strong local representation and are generally practical for moderate-sized dermoscopic datasets. Transformers offer broader contextual modeling but can require greater Pre-training data and computational resources. CNN-Transformer hybrids and ensembles can integrate complementary information but increase architectural and deployment complexity. The representative studies summarized in Table 5 illustrate these trade-offs across traditional machine-learning, CNN, Transformer, hybrid, ensemble, self-supervised, and multimodal approaches, with differences in dataset size, class distribution, computational requirements, interpretability, and validation design. Model selection should therefore consider dataset size and diversity, class distribution, computational constraints, intended clinical use, interpretability, calibration, uncertainty, and external validation. Benchmark accuracy alone is insufficient for determining clinical relevance. A model with slightly lower internal accuracy but better calibration, reproducibility, efficiency, and external validation may provide more useful evidence than a substantially more complex model evaluated only on an internal benchmark.

### 5.5 Self-supervised, multimodal and foundation model learning

Self-supervised learning obtains representations from unlabeled images before task-specific fine-tuning and can reduce dependence on expert annotation. Approaches include contrastive learning, masked-image reconstruction, consistency learning, relational learning, and teacher-student knowledge distillation. Wang Y. et al. (2023) demonstrated self-supervised knowledge distillation for lightweight skin lesion classification.

Multimodal learning combines dermoscopic images with clinical images or metadata such as anatomical site, age, sex, symptoms, or lesion history. Wang H. et al. (2025) investigated self-supervised learning using dermoscopic and clinical images, while Das et al. (2025) examined multimodal fusion. These approaches can provide complementary information but introduce missing modalities, unequal image quality, incomplete metadata, privacy, and demographic-shortcut risks.

Foundation models provide reusable representations and adaptable capabilities. MedSAM demonstrated promptable segmentation adapted to medical imaging (Ma et al., 2024). However, foundation and self-supervised models may inherit Pre-training biases and require domain-specific evaluation. Assessment should therefore include external datasets, subgroup performance, uncertainty, computational cost, and clinically relevant failure cases rather than benchmark performance alone.

### 5.6 Explainable artificial intelligence

Explainable Artificial Intelligence (XAI) can help assess whether predictions rely on lesion characteristics or potentially irrelevant image features. Common approaches include Grad-CAM, attention visualization, and SHAP. Ruga et al. (2026) combined deep and engineered features, ensemble learning, Grad-CAM, and SHAP in MultiExCam, while Fiaz et al. (2025) integrated lesion segmentation, multiclass classification, and Grad-CAM.

Visually plausible explanations do not necessarily demonstrate faithful model reasoning. XAI should therefore be assessed for faithfulness, stability, localization, clinical relevance, and usefulness. Explainability does not replace external validation, calibration, uncertainty assessment, or clinical oversight.

### 5.7 Current challenges in skin lesion classification

#### 5.7.1 Class imbalance

Skin lesion datasets commonly contain unequal representation of diagnostic categories, which can cause models to favor prevalent classes. Class weighting, focal loss, balanced sampling, minority-class augmentation, and synthetic data generation can mitigate this issue, but evaluation should include class-wise performance rather than overall accuracy alone. Bria et al. (2020) demonstrated the importance of addressing imbalance in medical-image detection, while Khatun et al. (2024) highlighted concerns regarding health inequalities when underrepresented populations, including darker skin tones, are insufficiently represented.

#### 5.7.2 Dataset bias, shortcut learning, and domain shift

Models may exploit rulers, color charts, hair, image boundaries, acquisition devices, background characteristics, or institution-specific processing rather than lesion pathology. Deployment data may additionally differ in camera type, dermoscope, illumination, resolution, institution, patient population, skin tone, lesion prevalence, and workflow. Such differences can produce strong internal performance but poor external generalization. Cross-dataset validation, artifact-focused testing, preprocessing controls, subgroup analysis, domain adaptation or generalization, and robustness testing under blur, noise, compression, occlusion, and unusual lesion appearances can help identify these limitations. Clinically meaningful population variation should not, however, be treated simply as unwanted distributional noise.

#### 5.7.3 Computational efficiency and clinical relevance

Large Transformers, ensembles, and hybrid architectures can increase memory and inference requirements. Claims of mobile or real-time use should report model size, parameters, computational operations, inference time, memory, hardware, and energy requirements. Knowledge distillation, pruning, quantization, and lightweight architectures may reduce these costs, but compression should not compromise sensitivity to malignant or rare lesions (Wang Y. et al., 2023). Clinical usefulness also requires evaluation according to the intended role, with calibration, uncertainty, subgroup analysis, workflow integration, privacy, and prospective or multi-center validation. Automated systems should support rather than replace clinical expertise.

### 5.8 Metrics for skin lesion classification

Classification performance should be evaluated using complementary measures. Accuracy summarizes overall correctness but can be misleading under class imbalance. Sensitivity measures detection of actual positive cases and is particularly important when missed malignant lesions may have serious consequences. Specificity measures the ability to correctly identify negative cases, while precision measures the proportion of positive predictions that are correct. F1-score balances precision and sensitivity, and balanced accuracy gives greater consideration to unequal class distributions.

AUC summarizes discrimination across classification thresholds, multiclass studies should specify whether class-wise, macro-averaged, micro-averaged, or one-versus-rest AUC is reported. Calibration evaluates whether predicted probabilities correspond to observed outcome frequencies and can be assessed using reliability diagrams, Expected Calibration Error, or Brier score. A highly discriminative model may nevertheless be poorly calibrated.

For robust reporting, studies should preferably provide overall and balanced accuracy, class-wise sensitivity and specificity, precision and F1-score, confusion matrices, class-wise or macro-averaged AUC, calibration, confidence intervals or statistical comparisons, external-dataset performance, subgroup results, and computational requirements when deployment is claimed.

## 6 Conclusion and future directions

### 6.1 Conclusion

This review shows that skin lesion analysis has progressed from handcrafted image processing and machine learning to CNNs, U-Net variants, Transformers, ensembles, self-supervised learning, multimodal methods, explainable AI, and foundation models. The evidence indicates that performance depends not only on architecture but also on dataset composition, preprocessing, class distribution, annotation quality, validation, computational cost, interpretability, and clinical context. CNN and U-Net methods remain practical for many dermoscopic datasets, whereas Transformer and hybrid models provide stronger global-context modeling but may require greater data and computation. Foundation and self-supervised methods may reduce annotation requirements, while multimodal learning can integrate complementary clinical information. However, these approaches may inherit dataset bias and domain shift. Explainability methods such as Grad-CAM and SHAP can support interpretation, but their faithfulness, stability, and clinical relevance require evaluation. Reported results also demonstrate why benchmark metrics are not directly comparable, as studies differ in datasets, partitions, preprocessing, architectures, and evaluation protocols. Key challenges remain class imbalance, dataset and device bias, limited skin-tone diversity, domain shift, inadequate external validation, uncertain calibration and explanations, and computational cost. Future progress should prioritize multi-center validation, reproducible patient-level evaluation, fairness, uncertainty-aware and interpretable models, efficient architectures, and evidence of clinical utility rather than isolated benchmark gains.

### 6.2 Future directions

#### 6.2.1 Diverse and multi-institutional datasets

Future datasets should include multiple institutions, devices, geographic locations, age groups, and skin-tone categories. Reporting should include acquisition device, dermoscope type, anatomical site, patient demographics, diagnostic confirmation, lesion prevalence, image quality, and whether images originate from the same patient or lesion, enabling patient-independent evaluation.

#### 6.2.2 External and prospective validation

External validation should use data not involved in training, model selection, hyperparameter optimization, or threshold selection. Cross-dataset testing can assess robustness to differences in devices, illumination, resolution, preprocessing, patient populations, lesion prevalence, and diagnostic practice. Prospective studies are needed to determine effects on clinical decisions, referrals, workload, and patient outcomes.

#### 6.2.3 Self-supervised learning

Self-supervised learning can exploit unlabeled dermoscopic and clinical images to learn domain-specific representations before supervised fine-tuning, potentially reducing annotation requirements. Future studies should compare self-supervised Pre-training with conventional transfer learning under equivalent conditions and report Pre-training data, learning objectives, augmentation, fine-tuning, external performance, subgroup results, and computational cost. Biases in unlabeled Pre-training data also require evaluation (Wang Y. et al., 2023; Wang H. et al., 2025).

#### 6.2.4 Medical Foundation-model adaptation

Foundation models may support segmentation, classification, retrieval, multimodal analysis, and annotation assistance. Research should investigate parameter-efficient fine-tuning, prompt learning, domain adaptation, few-shot learning, fairness, uncertainty, calibration, efficiency, and robustness. Evaluation should include small or indistinct lesions, rare categories, darker skin, hair occlusion, image-quality variation, and different acquisition devices. MedSAM demonstrates potential, but dedicated dermoscopic validation remains necessary (Ma et al., 2024).

#### 6.2.5 Explainable and uncertainty-aware systems

Future systems should combine clinically interpretable explanations with reliable uncertainty estimates. Grad-CAM, attention visualization, SHAP, prototype-based, concept-based, counterfactual, and example-based explanations should be assessed for faithfulness, stability, localization, and clinical relevance. Uncertainty methods should identify ambiguous or out-of-distribution cases and support appropriate referral or deferral. Calibration can be assessed using reliability diagrams, Expected Calibration Error, Brier score, or selective prediction (Ruga et al., 2026).

#### 6.2.6 Multimodal learning

Multimodal systems may combine dermoscopic images with clinical photographs, demographics, anatomical location, symptoms, lesion history, and other metadata. Research should address missing modalities, unequal image quality, incomplete metadata, and demographic or metadata shortcuts. Controlled ablation studies should determine the contribution of each modality (Wang H. et al., 2025).

#### 6.2.7 Domain adaptation and generalization

Training and deployment data may originate from different distributions. Future approaches should investigate feature alignment, adversarial adaptation, style transfer, domain-specific normalization, test-time adaptation, and domain generalization. Studies should distinguish methods requiring target-domain information from those designed to generalize without it and ensure that clinically meaningful population variation is not removed as unwanted domain variation.

#### 6.2.8 Continual and lifelong learning

Clinical systems may encounter new lesion classes, devices, patient populations, and clinical practices after deployment. Continual learning could support model updates, but catastrophic forgetting, mislabeled data, bias amplification, model drift, and regulatory challenges require version control, monitoring, auditing, rollback procedures, and periodic validation.

#### 6.2.9 Privacy-preserving and federated learning

Federated learning can enable collaborative model development without directly exchanging patient images. Future studies should address institutional heterogeneity, communication overhead, unequal contributions, privacy risks, poisoning attacks, governance, reproducibility, and liability. Federated and centrally trained models should be compared under equivalent conditions with institution-level performance reported.

#### 6.2.10 Efficient mobile and point-of-care systems

Lightweight architectures, knowledge distillation, pruning, quantization, and parameter-efficient adaptation may facilitate mobile and resource-limited applications. Deployment studies should report model size, parameter count, computational operations, inference time, hardware, memory, and energy requirements. Efficiency should not compromise sensitivity to malignant or rare lesions, and intended clinical use should be clearly defined.

#### 6.2.11 Standardized evaluation and reproducibility

Future studies should report complementary performance metrics, calibration, confidence intervals, computational requirements, and external validation. Reproducibility additionally requires patient-independent partitioning, detailed preprocessing and augmentation, training settings, random seeds, model checkpoints, statistical comparisons, and hardware information.

#### 6.2.12 Human-AI partnership

Automated skin lesion analysis should be investigated primarily as clinical decision support rather than assumed to provide autonomous diagnosis. Studies should determine whether model predictions, explanations, and uncertainty estimates improve diagnostic sensitivity, referral decisions, clinician confidence, workload, efficiency, and patient safety. Human-AI performance should be compared with human-only and model-only conditions.

#### 6.2.13 Regulatory, ethical, and workflow considerations

Clinical deployment requires attention to regulation, privacy, accountability, cybersecurity, data governance, and workflow integration. Future studies should define intended users, target populations, clinical settings, input-quality requirements, model outputs, uncertainty handling, responsibility for errors, update procedures, and post-deployment monitoring. Fairness should be assessed across relevant demographic and acquisition-related groups, with AI supporting rather than overriding clinical decision-making.

### 6.3 Most important research gaps

Based on the reviewed literature, the most important research gaps include:

1. Multi-center external and prospective clinical validation.
2. Increased diversity of skin tones, patient cohorts, and imaging acquisition environments.
3. Improved sensitivity to malignant and rare lesions.
4. Detection of and mitigation of dataset shortcuts, device bias, and domain shifts.
5. Faithful explanation generation and uncertainty quantification.
6. Evaluation protocols based on patient-level rather than image-level separation.
7. Reduction in the computational requirements of Transformer-, ensemble-, and foundation-model approaches.
8. Multi-institutional validation of self-supervised and foundation model-based methods.
9. Incorporation of multimodal clinical data while avoiding demographic and metadata shortcuts.
10. Reproducible preprocessing, training, and evaluation protocol development.
11. Requirements for regulatory, ethical, privacy, accountability, and post-deployment monitoring aspects.
12. Demonstration that collaborative human-AI systems lead to tangible clinical benefit.

Deep learning techniques have significantly enhanced skin lesion segmentation and classification. However, the leap from benchmarks to trustworthy clinical decision making requires simultaneous consideration of safety, robustness, fairness, interpretability, reproducibility, efficiency, and clinical utility.

## Glossary

AK: Actinic Keratosis
AUC: Area Under the Receiver Operating Characteristic Curve
BCC: Basal Cell Carcinoma
BKL: Benign Keratosis Lesion
CIELAB: Commission Internationale de l’Éclairage L*a*b* color space
CLAHE: Contrast-Limited Adaptive Histogram Equalization
CNN: Convolutional Neural Network
CRF: Conditional Random Field
DF: Dermatofibroma
FN: False Negative
FP: False Positive
GAN: Generative Adversarial Network
GLCM: Gray-Level Co-occurrence Matrix
HAM10000: Human Against Machine with 10,000 Training Images
HSV: Hue–Saturation–Value
IoU: Intersection over Union
ISIC: International Skin Imaging Collaboration
KNN: K-Nearest Neighbors
MEL: Melanoma
NV: Melanocytic Nevus
PH^2^: PH^2^ Dermoscopic Image Database
RF: Random Forest
RGB: Red–Green–Blue
SAM: Segment Anything Model
SCC: Squamous Cell Carcinoma
SHAP: SHapley Additive explanations
